# Soil and plant phytoliths from the *Acacia-Commiphora* mosaics at Oldupai Gorge (Tanzania)

**DOI:** 10.7717/peerj.8211

**Published:** 2019-12-11

**Authors:** Julio Mercader, Siobhán Clarke, Mariam Bundala, Julien Favreau, Jamie Inwood, Makarius Itambu, Fergus Larter, Patrick Lee, Garnet Lewiski-McQuaid, Neduvoto Mollel, Aloyce Mwambwiga, Robert Patalano, María Soto, Laura Tucker, Dale Walde

**Affiliations:** 1 Department of Anthropology and Archaeology, University of Calgary, Calgary, Canada; 2 Department of Archaeology, Max Planck Institute for the Science of Human History, Jena, Germany; 3 Department of Archaeology and Heritage Studies, University of Dar es Salaam, Dar es Salaam, Tanzania; 4 Department of Anthropology, University of Toronto, Toronto, Canada; 5 Tropical Pesticides Research Institute, National Herbarium of Tanzania, Arusha, Tanzania; 6 Arusha National Natural History Museum, Arusha, Tanzania; 7 ASM Research Group, Cochrane, Canada

**Keywords:** African palaeoenvironments, Soil and plant phytoliths, *Acacia-Commiphora* woodland and grassland mosaics, Reference collections, East Africa, Analog for early human evolution

## Abstract

This article studies soil and plant phytoliths from the Eastern Serengeti Plains, specifically the *Acacia-Commiphora* mosaics from Oldupai Gorge, Tanzania, as present-day analogue for the environment that was contemporaneous with the emergence of the genus *Homo*. We investigate whether phytolith assemblages from recent soil surfaces reflect plant community structure and composition with fidelity. The materials included 35 topsoil samples and 29 plant species (20 genera, 15 families). Phytoliths were extracted from both soil and botanical samples. Quantification aimed at discovering relationships amongst the soil and plant phytoliths relative distributions through Chi–square independence tests, establishing the statistical significance of the relationship between categorical variables within the two populations. Soil assemblages form a spectrum, or cohort of co-ocurring phytolith classes, that will allow identifying environments similar to those in the *Acacia*-*Commiphora* ecozone in the fossil record.

## Introduction

Multiple proxies indicate that during the last two million years the East African climate changed, triggering a shift in its plant landscape from forested ecosystems to open woodland/grassland mosaics. These proxies include paleosol carbonates (Cerling, Bowman & O’Neil, 1988; Levin et al., 2004; Quinn et al., 2007; Levin et al., 2011), vertebrates (Bibi & Kiessling, 2015; Bibi et al., 2018; Prassack et al., 2018), palaeobotanical remains (Bonnefille, 1984; Bonnefille, 1995), and stable isotopes (Magill, Ashley & Freeman, 2013a; Magill, Ashley & Freeman, 2013b; Uno, Polissar & Jackson, 2016; Lupien et al., 2018). This environmental transformation led to the widespread occurrence of a plant landscape characterized by *Acacia-Commiphora* woodland (White, 1983), and corresponds with key changes in hominin evolution and ecology (e.g., Ravelo et al., 2004; Lepre et al., 2011; Beyene et al., 2013).

Over the last few decades, different investigations have attempted to identify modern ecosystems as referential correlates for Pleistocene East African vegetation communities within the Somalia-Masai floristic region; a center of endemism that extends over 2 million km^2^ in parts of Ethiopia, Sudan, Uganda, Kenya, and Tanzania. For example, lakes such as Manyara and Makat are often considered correlates for paleo-Lake Oldupai as they have vegetation types not in equilibrium with the regional climate (Barboni, 2014; Copeland, 2007). Peters & Blumenschine (1995) developed a paleolandscape model that predicted possible hominin land use patterns based on reconnaissance surveys in eastern and southern Africa. Copeland (2007) examined habitats in northern Lake Manyara, Ngorongoro, and the Serengeti Plain as baseline to understand the paleolandscape inhabited by early *Homo* in terms of climate, land forms, and soil types.

At present, there is no extensive, ecosystem-wide phytolith analog for the *Acacia*-*Commiphora* landscape that was the backdrop for human evolution in some regions of East Africa over the last two million years (Bobe & Behrensmeyer, 2004; Plummer et al., 2009; Cerling et al., 2011; Blumenthal et al., 2017). Researchers have focused instead on a variety of microhabitats such as groundwater-fed woodlands (e.g., Ashley et al., 2010; Barboni et al., 2010), mosaic fluviatile systems (e.g., Arráiz et al., 2017), and lake-shore palm groves (Albert, Bamford & Cabanes, 2009; Albert, Bamford & Esteban, 2015).

In Quaternary palaeoecology, phytolith analysis has successfully addressed evolutionary and environmental questions (see review in Strömberg et al., 2018). Phytoliths are microscopic silicifications mirroring the morphometric characteristics of the plant organs where they precipitate, inside and between plant cells of varied groups (Rovner, 1971; Piperno, 1988; Hodson et al., 2005; Hodson, 2016; Strömberg, Di Stilio & Song, 2016). Phytogenic silica is released into soils after litter decomposes and discharges both amorphous silica and phytoliths. This process creates a synchronous phytolith record of the plant communities that grew in the soil (Piperno & Becker, 1996; Kerns, Moore & Hart, 2001; Clarke, 2003; Piperno, 2006; Thorn, 2006; Honaine, Zucol & Osterrieth, 2009; Morris, Ryel & West, 2010; Mercader et al., 2011; Novello et al., 2012; Novello et al., 2018; Blinnikov, Bagent & Reyerson, 2013; An, Lu & Chu, 2015; Watling et al., 2016; Esteban et al., 2017; Fick & Evett, 2018).

We explore the soil phytolith assemblages from *Acacia*-*Commiphora* woodlands to identify similar environmental contexts in the fossil record, undertaking a concurrent study of phytoliths from local plants to aid in taxonomic classification (Wallis, 2003; Carnelli, Theurillat & Madella, 2004; Gallego & Distel, 2004; Bremond et al., 2005; Tsartsidou et al., 2007; Barboni & Bremond, 2009; Mercader et al., 2009; Mercader et al., 2010; Gao et al., 2018a; Gao et al., 2018b). The comparison of soil and plant phytoliths can assist in tracking the boundary between woodlands and grasslands (Kerns, Moore & Hart, 2001; Morris, Ryel & West, 2010; Mercader et al., 2011). Elsewhere, this comparison has been helpful to assess phenomena such as time averaging (Alexandre et al., 1997; Blecker et al., 2006; Mercader et al., 2011; Hyland, Smith & Sheldon, 2013), catchment (Fredlund & Tieszen, 1994; Blinnikov, 2005), and ecological succession (Leakey, 1979; Niboye, 2010) that are hereby studied at the regional and local level.

## Study area

**Figure 1:**
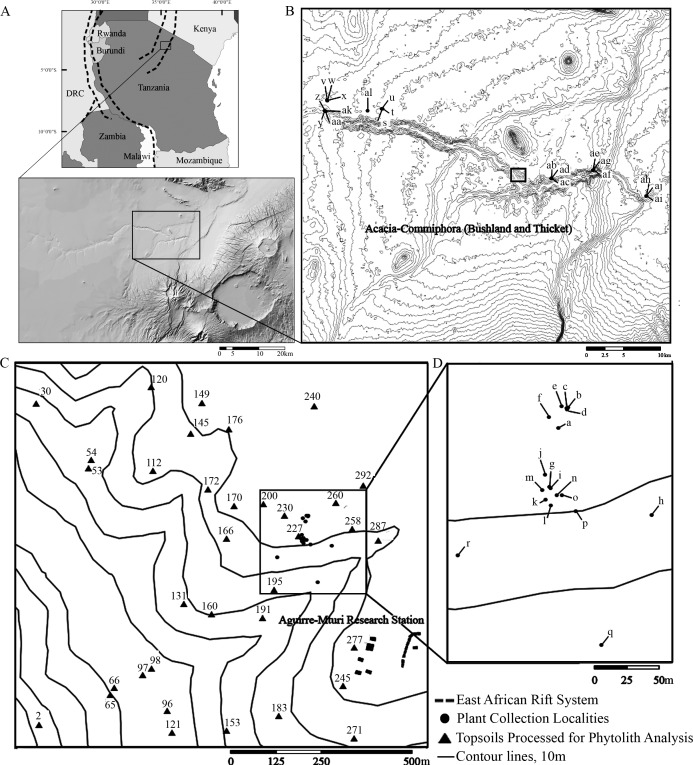
Location map of Oldupai Gorge. (A) Location map of Oldupai Gorge at the continental and regional levels; (B) Plant sampling localities within the Oldupai Gorge region and location of 100 ha transect; (C) 100 ha transect with the topsoil sampling localities and location of 6.25 ha quadrat; (D) 6.25 ha plant sampling localities. Plant sampling legend: (A) *Cynodon dactylon* (B) *Sporobolus panicoides* (C) *Asparagus africanus* (D) *Ocimum* spp. (E) *Acacia tortilis* (F–H) *Sansevieria robusta* (I) *Aristida adoensis* j*) Commiphora africana* (K) *Commiphora kua* (L) *Ximenia caffra* (M) *Acacia mellifera* (N) *Barleria eranthemoides* (O) *Salvadora persica* (P) *Aloe secundiflora* (Q) *Sarcostemma viminale* r*) Cissus cactiformis* (S) *Hypoestes forskaolii* (T–U) *Acacia tortilis* (V) *Balanites aegyptiaca* (W) *Commiphora* spp*.* (X) *Acacia tortilis* y) *Sporobolus consimilis* (Z) *Pennisetum mezianum* (AA) *Boscia angustiflora* (AB) *Ximenia caffra* (AC) *Commiphora merkeri* (AD) *Acacia mellifera* (AE) *Cissus quadrangularis* (AF) *Aloe secundiflora* (AG) *Asparagus africanus* (AH) *Balanites aegyptiaca* (AI) *Acacia mellifera* (AJ) *Ximenia caffra* (AK) *Maerua triphylla* (AL) *Acacia nilotica*. Map Source: 2017 ESRI ArcGIS Image Service.

Oldupai Gorge falls within the Northern Tanzania Divergence Zone along the East African Rift System (Dawson, 1992; Foster et al., 1997) (Fig. 1A). It represents the boundary between central Tanzania’s Archaean craton and the north-south trending Mozambique belt, a product of the neo-Proterozoic Pan-African orogeny (Holmes, 1951; Cahen et al., 1984). Oldupai Gorge sits on the western flank of the Gregory Rift in the Ngorongoro Conservation Area (2°59′46.87″S, 35°21′7.50″E) between the volcanic highlands to the south and east, the metamorphic complexes to the north, and the Serengeti Plains to the west. Paleolake Oldupai was part of an endorheic basin formed >2 Ma as a result of tectonic subsidence and concomitant uplift in the Ngorongoro Volcanic Highlands (Hay, 1976), where numerous volcanoes existed (Mollel & Swisher III, 2012). Oldupai Gorge boasts well-dated archaeological and fossil records (Leakey et al., 1972; Hay, 1976; Deino, 2012). Its sedimentary beds span the Pleistocene (Beds I–IV, Masek, Ndutu, and Naisiusiu) and situate two million years of evolutionary history, from the Oldowan to the Later Stone Age (Leakey, 1971; Leakey & Roe, 1994).

The Eastern Serengeti Plains (Fig. 1) offer a varied topography between 1,350 m–1,550 m above sea level with a central plateau around Oldupai Gorge and highlands toward Endulen, enduring wide temperature oscillations and high winds (Anderson & Talbot, 1965; EAMD East African Meteorological Department, 1967; Norton-Griffiths, Herlocker & Pennycuick, 1975; White, 1983). Annual rainfall ranges from 380 mm to 803 mm per year, but at the Gorge it fluctuates from 331 mm–531 mm (Herlocker & Dirchl, 1972). The dry months in Oldupai Gorge extend from June to October, with the wettest phase lasting from December to April. Rainfall patterns and localized soil conditions influence the vegetation communities observed in the Serengeti (Anderson & Talbot, 1965). The dominant soils are calcimorphic, yellowish brown, with low humic values (<1% organic carbon) and a hard pan (Anderson & Talbot, 1965).

## Materials and Methods

The Tanzania Commission for Science and Technology (permit 2018-112-NA-2018-36), and the Tanzanian Ministry of Natural Resources and Tourism, through its Antiquities Division (permit 14/2017/2018) approved this work. Authorities at the Ngorongoro Conservation Area allowed us to enter the protected area (BE.504/620/01/53). The export license for the materials presented in this study were obtained from the Antiquities Division (EA.150/297/01: 5/2018/2019) and the Tanzanian Executive Secretary from the Mining Commission (00001258).

### Vegetation survey

The greater Serengeti area falls within the Somalia-Masai floristic region in which *Acacia*-*Commiphora* deciduous bushland is the climax vegetation (Kindt et al., 2011). This ecoregion is transitional to miombo woodlands to the south (Frost, 1996) and the Zanzibar Inhambane coastal forest mosaic to the east (Lovett & Wasser, 1993). The published plant inventory for Oldupai Gorge totals 32 genera (Herlocker & Dirchl, 1972). We studied 29 species representing 20 of these genera and 15 families. Characteristic Somalia-Masai taxa analysed here comprise the dominant members of the canopy (*Acacia mellifera*, *A*. *nilotica*), emergents (*Boscia angustifolia*), bushes (*Barleria eranthemoides*, *Maerua triphylla*), the herbaceous layer (*Hypoestes forskaolii*, *Ocimum* spp.), succulents (*Sansevieria robusta*, *Aloe secundiflora*), and the grasses (*Sporobolus consimilis*, *Cynodon dactylon* -Chloridoideae, *Aristida adoensis -* Aristidoideae).

Vegetation in the greater Oldupai region has been monitored for decades (Niboye, 2010). The average perennial cover today is 22% (Niboye, 2010), which is comparable to that observed several decades ago (Anderson & Talbot, 1965), with bare terrain exposing >45% of the area. Herlocker & Dirchl (1972) noticed an increased plant variety in the eastern sector of the gorge, introduced by the topographic changes from the canyon walls, and thus a higher diversity of bushes and grasses. They also noticed commonalities with the communities from the western gorge.

Ecologically, the plant community that typifies the natural vegetation at Oldupai Gorge is short *Acacia* and *Commiphora* woodland. Several tree species from these two genera co-dominate along with bushes such as *Barleria eranthemoides*. Succulents abound in ravines and depressions where several species of *Sansevieria*, *Euphorbia*, and *Sarcostemma* are common. Herbaceous plants are variably scattered across the landscape but, overall, they are low in numbers. Importantly, this domain is characterized by a paucity of grass taxa, which are typically inconspicuous and occasional (Kindt et al., 2011).

At the regional level (Fig. 1B) the vegetation was surveyed using qualitative criteria, aimed at investigating the range of species present in the greater Oldupai region, learning about their phytolith production and morphotype characteristics, and comparing the botanical dataset with the soil group. We did not attempt to collect plant specimens in numbers proportionate to frequency in each of their respective ecological communities, nor to other physiognomic variables or phylogenetic affiliation. Instead, we collected representative plants from the western and eastern gorge at the end of the wet season into the dry season in 38 sampling locations to target 29 species along an E-W transect (25 km long) from Granite Falls to Olbabal. Field samples were identified in the National Herbarium of Tanzania at the Tropical Pesticides Research Institute. When species level identification was uncertain and could belong to several species within the genus, we listed it as spp. Grouping and nomenclature come from the index of accepted names for the flowering plants of Sub-Saharan Africa (Klopper et al., 2006).

At the local level, the vegetation was surveyed using quantitative criteria. First, an aerial image covering 1 km^2^ (Fig. 1C) taken at the end of the wet season in the eastern half of Oldupai Gorge, was georeferenced via ArcGIS - ArcMAP 10.5.1. A grid consisting of 100 cells, each 100 m^2^, was superimposed. The cover-abundance value was digitized in each cell as a polygonal shape file, and classified it in four classes or ranks:

1) Rank zero, or ‘No-cover’: when plant cover constituted <10% within the spatial polygon (45.64% of the total area).

1) Rank one, or ‘Sparse’: when plant growth was 10%–20% (20.32% of the total).
2) Rank two, or ‘Open’: when plant growth was 20%–60% (26.41% of the total).
3) Rank three, or ‘Closed’: when plant growth was >60% (7.63% of the total).

**Figure 2:**
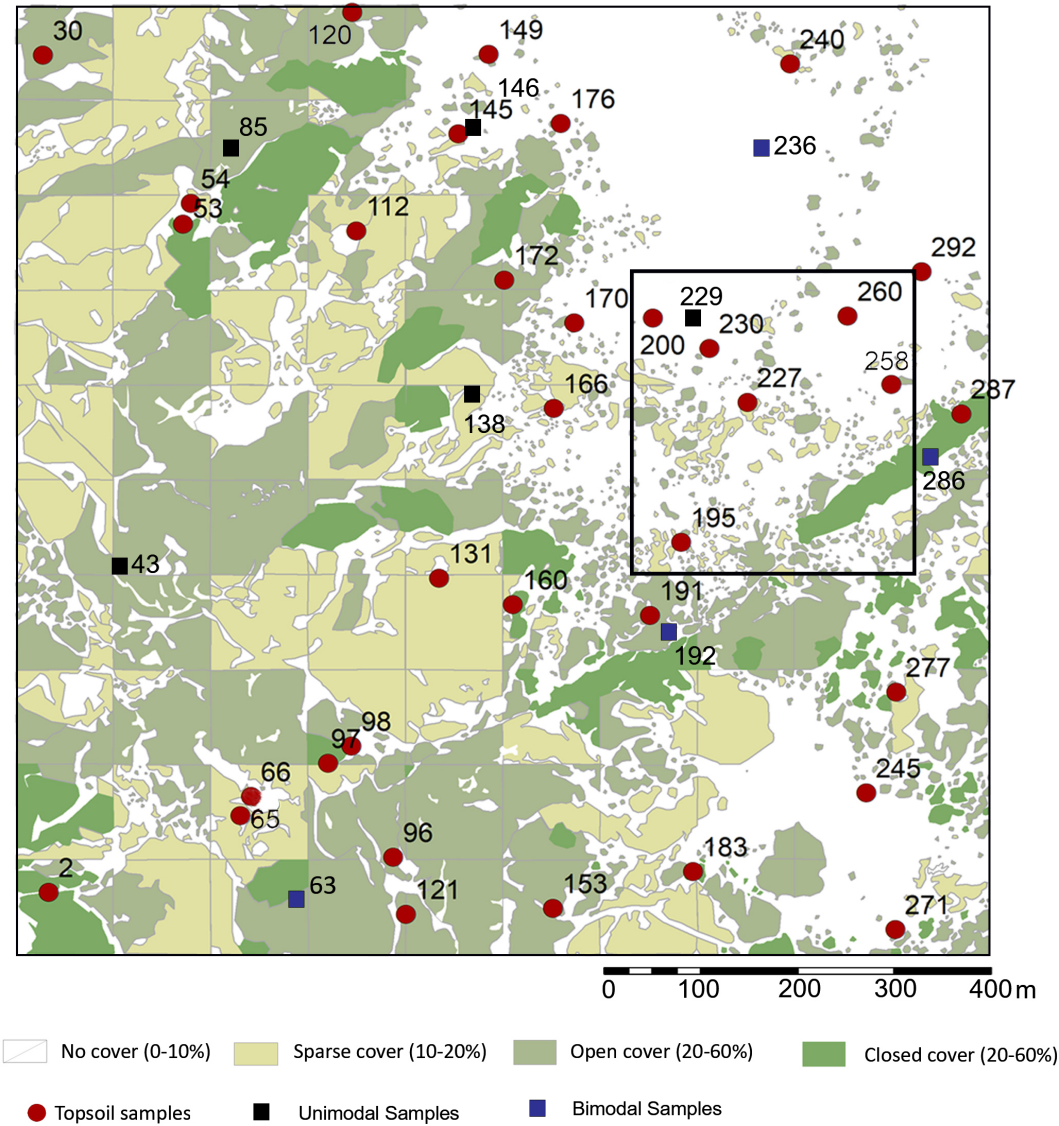
Sampling localities for soils and particle size analysis in relation to plant cover rank, with catchment transect in outline. See Table 1 for analytical description of soils. Digitized source: 2017 ESRI ArcGIS.

This rank system was subjected to groundtruthing in 300 spots (Mercader et al., 2017: Fig. 2), and then we proceeded to conduct a qualitative vegetation survey of the entire 1 km^2^ plot (Fig. 1C). After this, we zoomed into a 62,500 m^2^ quadrat (Fig. 1D) to conduct a full inventory, plotting, and collection of all plants present within this smaller plot. According to plant cover rank (Fig. 2), thirteen samples (37.14%) were recovered from rank zero; eight (22.86%) from rank one; eight (22.86%) from rank two; and six (17.14%) from rank three. Samples were each assigned to one of two topographical categories: promontory or ravine. Ravines are at a mean elevation of 1,422.66 m with a range between 1,412 m and 1,441 m (29 m). Promontories are at a mean elevation of 1,452.41 m with a range between 1,416 m and 1,469 m (53 m). Most samples (*n* = 29, 82.86%) were recovered from promontory locations (Fig. S1).

### Particle size analysis

We carried out particle size analysis of selected soil samples (*n* = 10) via laser diffraction (Malvern Mastersizer 2000: Sperazza, Moore & Hendrix, 2004). A soil sample (1.0 g) was deflocculated with 0.1% sodium hexametaphosphate. Ultrasonics dislodged clays for 5 min. Sample was left on orbital shaker overnight at 200 rpm. We cleansed the sample of carbonates, phosphate, and organics via hydrochloric acid (HCl 37%), nitric acid (HNO_3_ 60%) and hydrogen peroxide (H_2_O_2_ 30%) successively, rinsing the sample three times via centrifuging in between each treatment (3,000 rpm, 5 min). When measuring particle size in the Malvern 2000, we used obscuration rates above 15% but below 30%. The measurement of pH in all soils was through a ThermoFisher Orion 320 PerpHecT LogR meter, calibrated daily. The Ag/AgCl electrode was rinsed after calibration and in between samples. Sediment color was estimated dry under constant light conditions by two observers relative to the Munsell color system. Water loss on drying is the balance between soil field moisture in three grams of soil and the mass of the sample after drying at 110 °C for 24 h.

### Soil phytoliths

Sampling was never conducted on archaeological sediments, nor within any of the well-known and mapped lacustrine and fluviatile outcrops that constitute the ancient beds (Leakey et al., 1972; Hay, 1976); our work was restricted to purely surficial sandy loam (Anderson & Talbot, 1965). Prior to collecting soil samples, and to better understand local soils and visualize their horizons, several trenches were dug reaching the C-horizon: a characteristic underlying hard pan described by earlier soil analysts (Anderson & Talbot, 1965; Jager, 1982). In section, we observed a thin A-horizon, restricted to the rhizosphere and litter zone, while a B-horizon seems absent. We scraped 25 cm^2^ off the surface (5 ml) to a maximum depth of one cm within the A-horizon (Fishkis, Ingwersen & Streck, 2009; Mercader et al., 2010), excluding litter and not mixing or generating composite samples. Thirty-five loci were selected for stratified sampling, so that a comparable number of samples were collected from each plant cover type. The median distance between all soil assemblages is 516 m (measured distances, *n* = 522) over a range of 13 m–1,252 m. Geographical coordinates were documented as point features. The full dataset of soil samples including provenance, elevation, coordinates, processing weights, and individual counts are available at the Federated Research Data Repository (DOI https://dx.doi.org/10.20383/101.0122).

Detailed phytolith extraction protocols are available with the Federated Research Data Repository (DOI https://dx.doi.org/10.20383/101.0123), which are modified from (Albert et al., 1999). In brief, extraction from sediment used a 3.0 g aliquot that received 10 ml of 0.1% sodium hexametaphosphate for clay dispersion, followed by sonication (15 min) and overnight orbital shaking plus decantation of clays. Samples were rinsed and dried over two days (>70 °C). Soil samples received 10 ml equal parts 3N hydrochloric and nitric acids; then, rinsed and centrifuged at 3,000 rpm. Drying followed for two days (70 °C). Samples were weighed and transferred to Petri dishes on a hot plate (70 °C) whereby 10 ml of hydrogen peroxide (30%) was added to remove organics. Rinsing, drying, and weighing followed. Phytolith separation was done by transferring this fraction to 15 ml centrifuge tubes, to which 5 ml sodium polytungstate (∼2.4 s.g.) was added. Samples were vortexed for five minutes at 3,000 rpm. The supernatant was removed. A gradual decrease in specific gravity over the course of three subsets (subfractions 1, 2, 3) concentrated phytoliths in the third extract. Samples were rinsed with de-ionized water before centrifuging twice more, followed by two cycles of high centrifugation (4,500 rpm). Samples dried at 70 °C another time and a final weight obtained. The aliquot extracted for phytolith analysis weighed approximately 0.001 g. It was mounted with Entellan New and inspected while fresh; allowing particles to be rotated in three dimensions under the microscope. Phytoliths from soil samples were counted on one slide over 600 mm^2^ to the total amount, reaching average counts of 250. Diatoms were tallied concurrently. Carbonate content was calculated as the loss on acid treatment relative to the original mass. Extraction weights are the balance between the original mass and that of the extract after defloculation, carbonate removal, destruction of phosphate, elimination of organics, rinsing, and drying.

### Plant phytoliths

Extraction followed Albert & Weiner (2001), with modifications. Botanical specimens were sorted into anatomical parts, and each was individually weighed and measured. Full datasets with species, plant part studied, provenance, elevation, coordinates, processing weights, and individual counts are available at the Federated Research Data Repository (DOI https://dx.doi.org/10.20383/101.0122). All samples were cleansed by ultrasonication, then rinsed and dried overnight >80 °C. Dry weight was obtained, and samples placed in a muffle furnace (24 h, 400 °C). The resulting ashes were weighed and transferred to Petri dishes to which 10 ml of 3N hydrochloric and nitric acids were added to a hot plate at 70 °C for 30 min. Samples were transferred to 15 ml tubes and rinsed of remaining acids in three wash and centrifugation cycles at 3,000 rpm, with excess water being boiled off. Hydrogen peroxide (10 ml, 30%) was added to the Petri dishes heated on hot plates (70 °C), transferred to 15 ml centrifuge tubes, rinsed, and centrifuged (3,000 rpm) for three cycles. Samples were dried >70 °C overnight, and then weighed. An aliquot of approximately 0.001 g was analysed after mounting the sample for microscopy with Entellan New. Inspection captured three-dimensional characteristics during rotation conducted within 48 h of mounting and before the resin dried. Frequent polarization of the field of view under the microscope ensured that no anisotropic particles and crystals were confused for phytoliths. Neither fresh nor weather volcanic glass could be observed. We counted one full slide and only discrete phytoliths (average = 106 phytoliths), while articulated shapes were counted as one. As for very rich extracts, we tallied a minimum of 200 phytoliths and/or short cells for the grasses.

### Phytolith classification

There are two approaches to environmental reconstruction in phytolith research: the indices (Barboni, Bremond & Bonnefille, 2007; Bremond et al., 2008) and the general approach (Strömberg, 2004). Different researchers adopt one approach or another depending on their work’s practice and scope. We followed the general approach here to detect plant variations at the spatial level. Quantitative analysis involved exploration aimed at discovering relationships amongst the soil and plant phytoliths, which, when combined and filtered, isolated groups, classes, and morphotypes unique to each set and those in common. Further investigation involved comparison of phytoliths from soils and plants to explore their relative distributions through Chi–square independence tests, establishing the statistical significance of the relationship between categorical variables within the two populations using a 95% confidence value (*p* = 0.05).

Two system, polarizing microscopes (Olympus BX51, Motic BA410E) allowed us to confirm the opal silica’s isotropy and tally phytoliths at 400x, following the International Code for Phytolith Nomenclature 1.0 to name and describe specimens (Madella, Alexandré & Ball, 2005), with exceptions duly noted. Geographically pertinent reference collections and published soil phytolith assemblages were consulted (Albert, Bamford & Cabanes, 2006; Albert, Bamford & Esteban, 2015; Bamford, Albert & Cabanes, 2006; Fahmy, 2008; Mercader et al., 2009; Mercader et al., 2010; Mercader et al., 2011; Barboni & Bremond, 2009; Eichhorn, Neumann & Garnier, 2010; Novello et al., 2012; Collura & Neumann, 2017; Neumann et al., 2017). Full datasets with individual counts for each morphotype are available at the Federated Research Data Repository (DOI https://dx.doi.org/10.20383/101.0122). We classified discrete phytolith shapes in four large groups (woody dicot, grass short cells, generic Poaceae, and rare) and 17 classes that encompass 76 types. The woody dicot group totalled the blocky class (Mercader et al., 2009; Novello et al., 2012; Collura & Neumann, 2017), cylindrical types from bark (Mercader et al., 2009), varied globular phytoliths (Runge, 1999; Mercader et al., 2009), tabular types from woody dicots (Mercader et al., 2009; Collura & Neumann, 2017), clavates (Mercader et al., 2009), and sclereid phytoliths (Collura & Neumann, 2017). Phytoliths from Poaceae short cells were subdivided in three classes: lobate (Fahmy, 2008; Neumann et al., 2017), rondels (Mercader et al., 2010; Novello et al., 2012; Neumann et al., 2017) and chloridoid saddles (Twiss, Suess & Smith, 1969; Mercader et al., 2010). Two additional groups comprised generic grass phytoliths and rare morphotypes.

It should be noted that the tabular class, with 12 shape variants, contains a confuser morphotype called here tabular sensu stricto, and defined as a narrow, thin parallelepiped shape with straight edges that may occur in both monocots and dicots. This confuser morphotype differs from the tabular psilate observed in woody dicots (this study) in that the latter is a wide and thick tabular shape with a very slight sinuosity to the outline.

## Results

### Soil phytoliths

#### Soil characteristics and phytolith abundance

**Figure 3:**
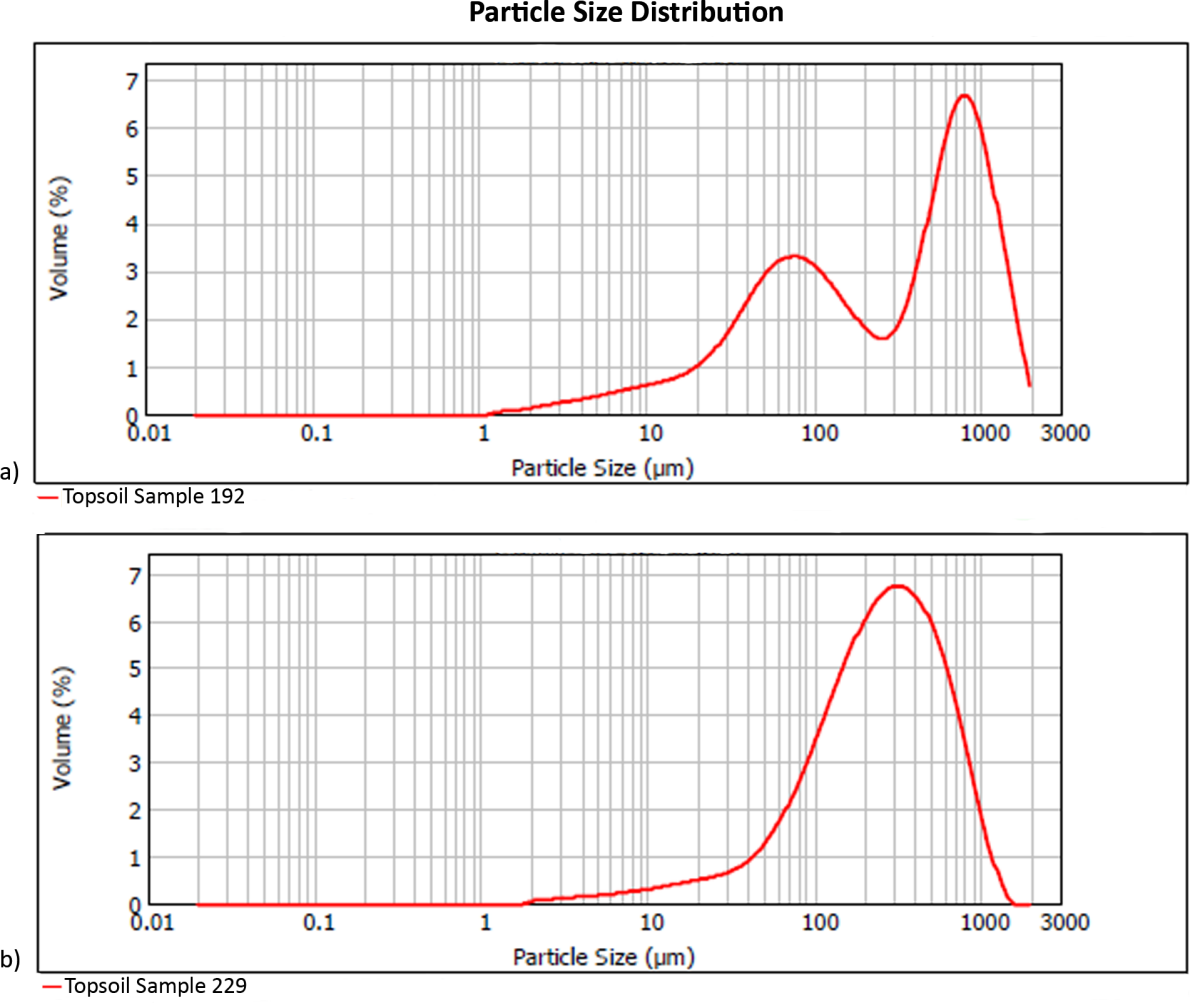
Particle size distribution examples (A) bimodal (Sample 192), and (B) unimodal (Sample 229). Refer to Table 1 for complete sample characterization, and Fig. S1 for sampling location. Digitized 2017 ESRI ArcGIS.

**Figure 4:**
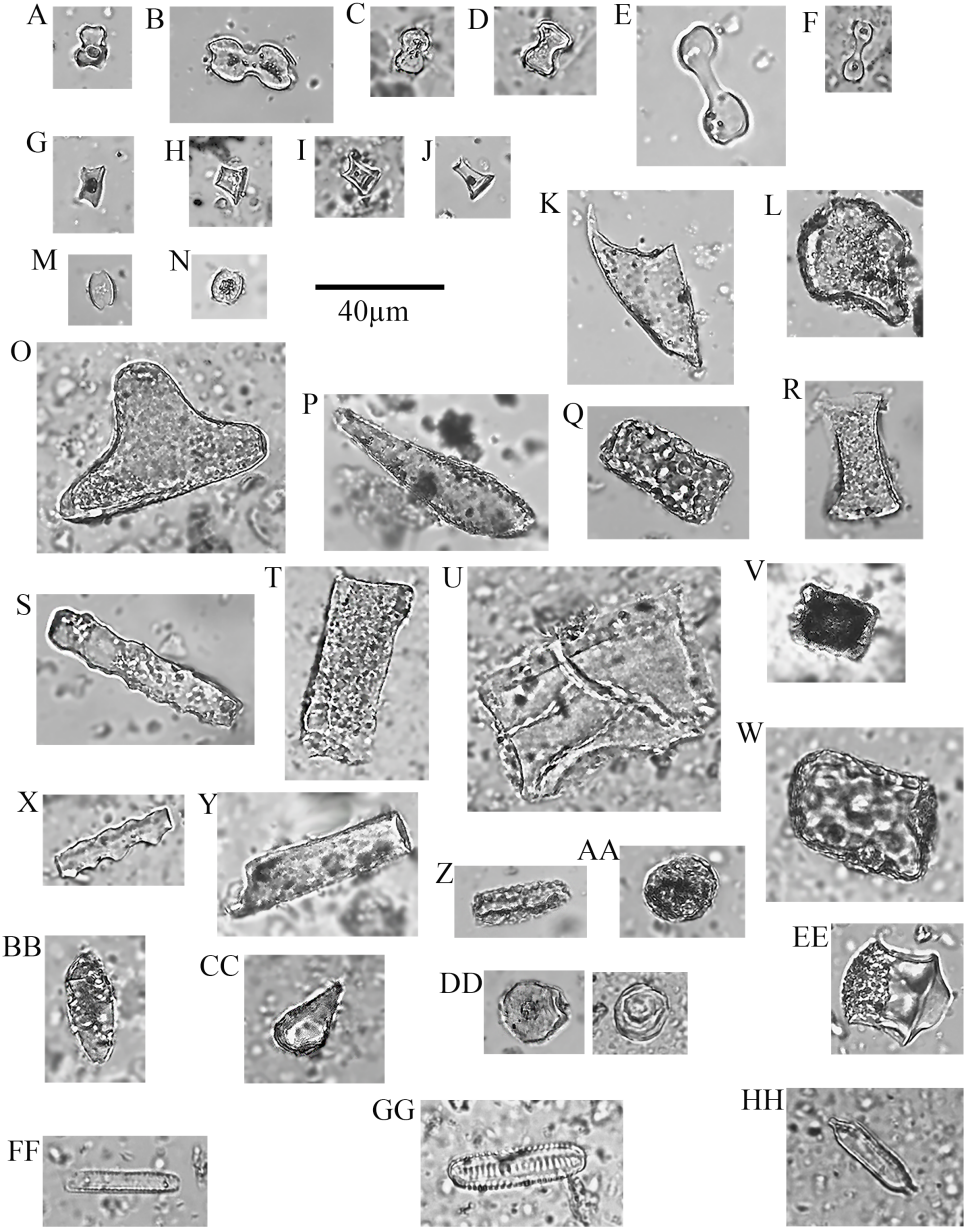
Soil phytoliths and diatoms. (A) bilobate, short concave (#30); (B) bilobate, short notched (#166); (C) bilobate short convex (#54); (D) bilobate, short flat (#112); (E) bilobate, long concave (#145); (F) bilobate, long convex (#98); (G) rondel (#30); (H) tower (#112); (I) tower, horned (#112); (J) tower, tapered (#65); (K) lanceolate (#30); (L) bulliform (#54); (M) saddle, squat (#30); (N) saddle (#65); (O) sclereid (#191); (P) clavate (#65); (Q) tabular thick lacunate (#54); (R) tabular strangulated (#127); (S) tabular sinuate (#120); (T) tabular scrobiculate (#65); (U) tabular sulcate (#30); (V) blocky (#30); W) blocky thick lacunate (#2); (X) cylindroid sinuate (#65); (Y) cylindroid scrobiculate (#149); (Z) cylindroid thick lacunate (#30); (AA) globular granulate (#191); (BB) guttiform (#30); (CC) fusiform (#54); (DD) globular facetate (#30); (EE) polygonal prism (#131); (FF–HH) diatoms (#65, 112, 258).

Local topsoils (Table 1) have a mean particle size of 344 µm, with pH of 8.7 and 5% water content. Unimodal particle size distribution is apparent in the poorly sorted soil matrices of most samples, while the coarse sand component in samples from the eastern half of our plot generates a bimodal population in which the poorly sorted fines represent the soil, and the well-sorted, coarse sands originate from easterly aeolian import (Fig. 3). Silt and very fine sands average 34% per sample, while carbonates are 69% by mass. The extracted phytolith-bearing fraction, which includes both biogenic and geogenic silica, is 54.53% (*M* = 53.85%; range = 45.54%, 25.23% –70.77%). Dissolution and mechanical breakage were rarely observed, and caution must be used not to confuse the effect of dissolution causing holes randomly through a phytolith versus the cavate textures that heavily decorate some blocky and tabular morphotypes (Figs. 4Q,  4W) from woodland taxa (cf. Mercader et al., 2009: Fig. 6g,k,l,m).

**Table 1:** Particle size analysis of selected topsoils, textural classification, phytolith-bearing fraction, modality, pH, color, and water content.

Sample	Mean particle size, µm		Particle class	Silt & Very Fine Sand, %		Modality	Sediment pH		Sediment colour		Sediment H_2_O content, %
43	294.2		Medium sand	39.62		Unimodal	8.7		Dark yellowish brown		3.9
63	513.8		Coarse sand	22.73		Bimodal	8.3		Moderate yellowish brown		5.4
85	216.9		Fine sand	49.49		Unimodal	8.8		Dark yellowish brown		6.5
138	94.7		Very fine sand	72.86		Unimodal	8.7		Dark yellowish brown		3.6
146	335.8		Medium sand	38.33		Unimodal	9.1		Pale yellowish brown		3.7
192	469.6		Medium sand	37.59		Bimodal	8.6		Moderate yellowish brown		2.7
229	322.1		Medium sand	26.31		Unimodal	8.7		Dark yellowish brown		2.8
236	670.3		Coarse sand	17.75		Bimodal	8.4		Dark yellowish brown		3.7
279	736.8		Coarse sand	6.54		Unimodal	9.2		Dark yellowish brown		2.9
286	300.1		Medium sand	35.52		Bimodal	8.6		Dark yellowish brown		4.0
Mean	344.3		Medium sand	29.55		Unimodal	8.7		Yellowish brown		3.8

**Figure 5:**
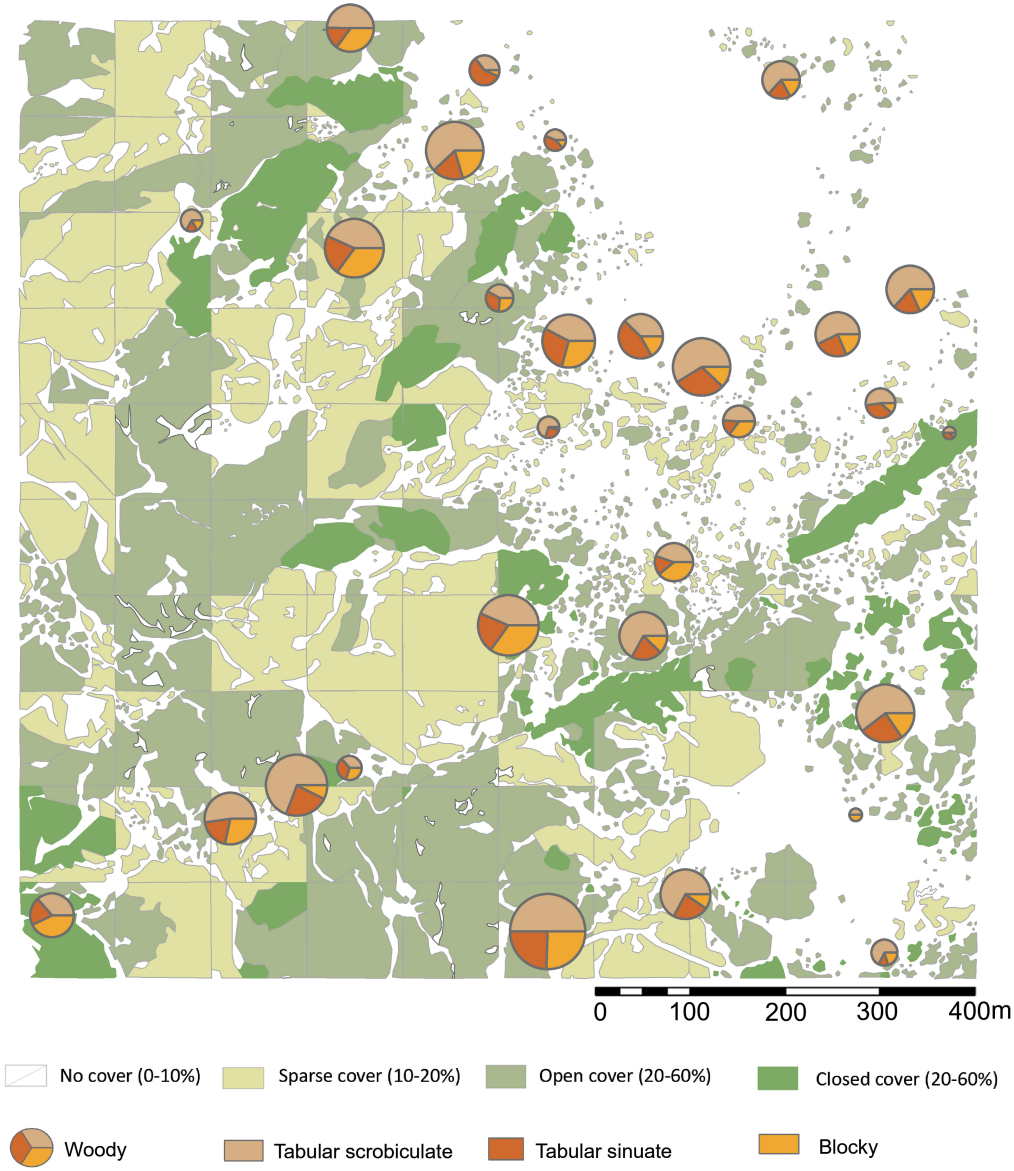
Highest ranking woody phytolith proportions in relation to plant cover rank. Please see Figs. 1 and 2 for location of sampling area and corresponding sample numbers. Digitized 2017 ESRI ArcGIS.

**Figure 6:**
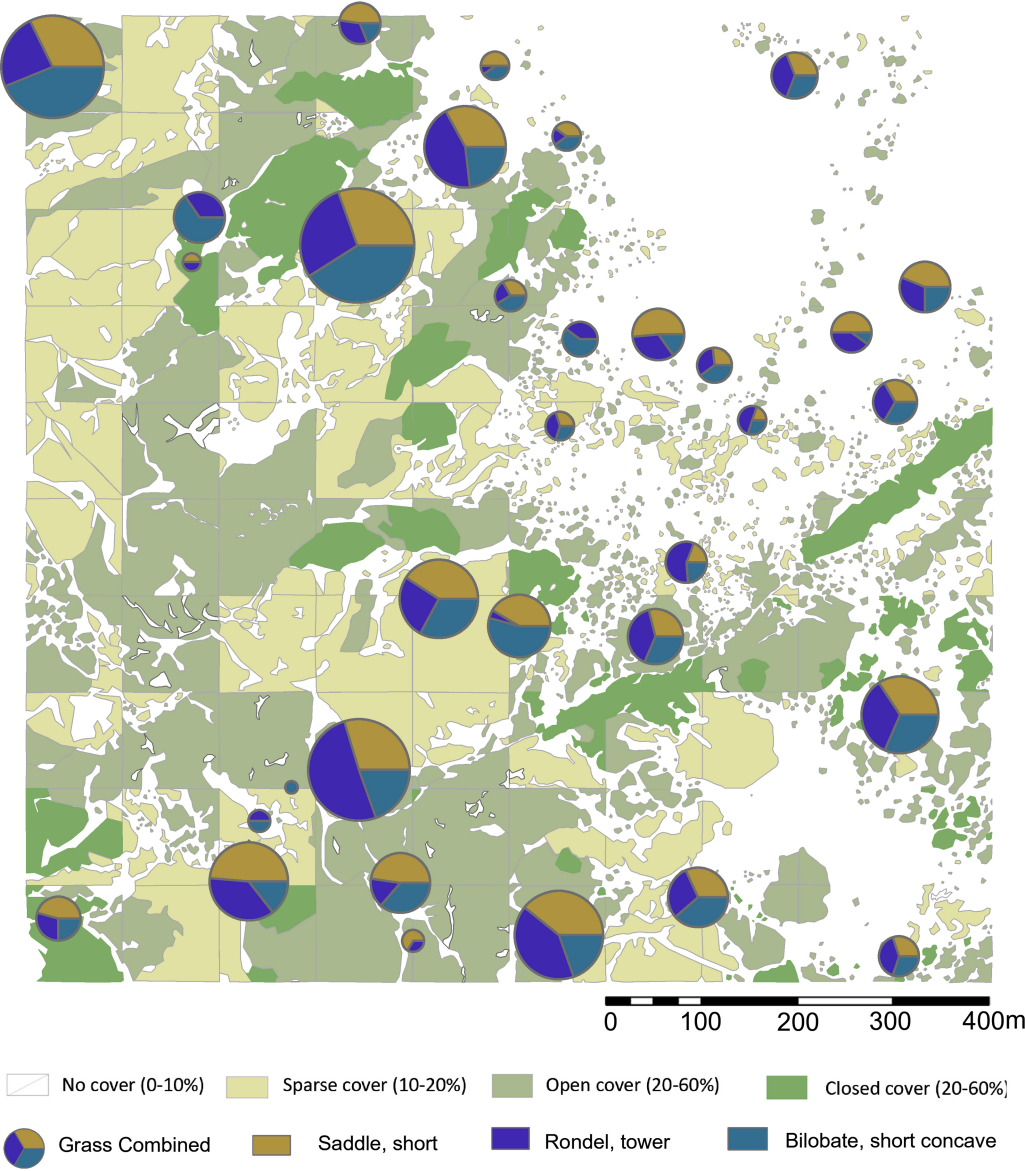
Highest ranking short cell phytolith proportions in relation to plant cover rank. Digitized 2017 ESRI ArcGIS. Please see Figs. 1 and 2 for location of sampling area and corresponding sample numbers. Digitalized Source: Google Maps.

No soil sample was void of phytoliths. In total, 10,745 phytoliths were tallied ($\bar {X}=$307, *M* = 285 per sample; range = 839, 16–855) containing 64 morphotypes that can be grouped into 15 major classes (Fig. 4, Table 2). Of these, six are prominent classes (Figs. 5, 6) and account for 83.78% of all soil phytoliths: tabular: 41.74%, blocky: 13.11%, globular: 8.52%, rondel: 6.98%, cylindric: 6.96%, and lobate: 6.47%. Within the tabular class, the three most prominent contributors represent 82.16% of the subtotal: scrobiculate: 45.40%; sinuate: 20.47%; and thick lacunate: 16.30%. Two types of blocky phytoliths represent 91.12% of the class; blocky: 62.38% and ridged: 28.74%. One single type of globular phytolith represents 85% of all globular types: a globular to hemispherical, facetate body with a centric cavity; possibly from mesophyll cells found in the leaves from several taxa in the Lauraceae (Figs. 4A and 4D; Thorn, 2001; Murungi, 2017). In contrast, the classic indicator of arboreal cover in the African tropics, the globular granulate, amounts to only 9% of the globular class. Two globular morphotypes were recovered from soils only: globulose bisected and segmented. Within the cylindric class, two phytoliths reach 91.44%: scrobiculate, 76% and sinuate, 15.37%.

Two types dominate the rondels (97.33%): Towers, 63.33% and rondels *sensu stricto*, 34%. Lastly, for the lobate class, 86.33% of the variability is represented by small bilobates with concave ends (61.58%), small bilobates with flat ends (16.04%), and small bilobates with convex ends (8.34%). Four lobate types were found exclusively in soils: small bilobate with notched ends, large bilobate with flat ends, large bilobate with notched ends, and cross. The saddle class is uncommon (5.87%) and the short saddle dominates it (74.32% of the subtotal), while squat saddles are rare. Generic tabulars (3.88% of the total), bulliforms and lanceolates (2.89% of the total), generic cylindric phytoliths (1.23% of the total), sclereids (1.1% of the total), fusiforms / gutiforms (0.74% of the total) (nomenclature not per ICPN 1.0), and clavates (0.25% of the total) are scarce. The remainder includes composite epidermal tissue, polygonal prisms with subrounded top (cf. *Commelinaceae*), and papillae; and they all are extremely uncommon.

**Table 2:** Topsoil phytolith counts per group, class, and morphotype.

**Major group**	**Phytolith class**	**Phytolith morphotype**	**Count**
Poaceae short cell	Lobate	Bilobate, long concave	38
		Bilobate, long convex	8
		Bilobate, long flat	14
		Bilobate, long notched	4
		Bilobate, short concave	428
		Bilobate, short convex	58
		Bilobate, short flat	114
		Bilobate, short notched	28
		Cross	1
		Polylobate	2
	Rondel	Rondel, horned	8
		Rondel, truncated	12
		Rondel, tower	475
		Rondel	255
	Saddle	Saddle, ovate	24
		Saddle, short	469
		Saddle, squat	140
	Blocky	Blocky	879
		Blocky corniculate	2
		Blocky ridged	405
		Blocky scrobiculate	23
		Blocky sinuate	28
		Blocky thick lacunate	72
	Cylindrical	Cylinder corniculate	3
		Cylinder psilate	132
		Cylinder scrobiculate	569
		Cylinder sinuate	115
		Cylinder thick lacunate	61
	Fusiform	Fusiform	11
		Guttiform	68
	Sclereid/Clavate	Clavate	27
		Sclereid	119
	Spherical	Globular bisected	1
		Globular facetate	778
Woody		Globular granulate	83
		Globular granulate, large	3
		Globular granulate oblong	24
		Globular psilate	10
		Globulose	2
		Globulose segmented	1
		Oblong granulate	10
		Hemisphere psilate	3
Tabular	Tabular corniculate	11
		Tabular ellipsoidal	35
		Tabular ellipsoidal, large	21
		Tabular facetate	8
		Tabular laminate	186
		Tabular oblong	16
		Tabular pilate	3
		Tabular scrobiculate	2036
		Tabular sinuate	918
		Tabular strangulated	11
		Tabular sulcate	509
		Tabular thick lacunate	731
Undetermined grass	Grass, undetermined	Bulliform	47
		Trichome	264
Rare/Unknown	Composite	Epidermal	3
		Vessel	18
	Drum	Polygonal prism	2
	Sedge	Papillae	1
	Short cell, other	Trapeziform sinuate	1
	Tabular undetermined	Tabular	70
		Tabular crenate	36
		Tabular psilate	311

Soils also produced 1,564 diatoms in addition to the phytolith complement summarized above. The mean number of diatoms per sample is 44.68 and the median recovery is 9.50 with a range of zero –301. The specimens are mostly from the Nitzschioid group (Figs. 4af–4ah), which grows in wet soils. The diatom count per plant cover class is inversely proportional to density of plant cover today, with the highest frequency in soil samples without vegetation and/or sparse growth probably because of pooling water in these areas (Albert, Bamford & Cabanes, 2006).

#### Extraction weight and plant cover

Conventionally, extraction weights are reported as a proxy of phytolith concentration. Like others before us (Blinnikov, Bagent & Reyerson, 2013), however, we did not see a discernible pattern between plant cover, phytolith count, and extraction weight (Fig. S1) with values randomly distributed. Thus, plant cover rank zero varied between 43.6% –69.5%, for rank one between 40.96% –70.77%, rank two between 25.23% –63.56%, and rank three between 41.1% –56.16%. The scores for plant cover ranks overlap, and so do those for extraction weight versus terrain elevation.

We explored the relationships between plant cover rank and phytoliths from woody dicots versus grasses. The woody phytolith group dominates all terrain ranks, and appears to increase with denser plant cover, while grass phytoliths peak in sparsely vegetated terrain. The null hypothesis posits that if plant cover has no influence on the relative proportions of woody and grass phytoliths, there will be no significant difference between overall proportions and plant cover rank proportions. For plant cover rank zero, vegetation density has not significantly influenced the relative proportions of woody and grass phytoliths present in the topsoil (Chi–square = 2.382, not significant at *p* < 0.10). There are significant differences in all other plant landscapes: Rank one has significantly more grass phytoliths than expected from the null hypothesis (Chi–square = 9.0812, *p* < 0.01) while ranks two (Chi–square = 7.2288, *p* < 0.01) and three (Chi–square = 8.0625, *p* < 0.01) have many more woody phytoliths. Statistically, therefore, sparse, open, and closed terrain influenced the relative proportions of woody and grass phytoliths released into the soil (Table 3), with sparse land containing more grass phytoliths, and open woodland / closed terrain supporting more woody dicot types.

For short cell classes (Table 4), rank zero yields rondels followed by lobates and saddles; rank one: lobates, saddles, and rondels; rank two and three: lobates, rondels, and saddles. These class fluctuations across plant cover, however, are artifacts of differing sample numbers per plant zone (Figs. S2–S4). Chi-square testing revealed no significant changes in short cell class related to plant cover rank. By morphotype, the most frequent phytoliths are towers, short saddles, and small bilobates with concave ends. In ranks zero and one, this order is preserved, but it reverses in ranks two and three. That is, the tandem of towers and short saddles are more frequent in areas of sparse to no plant cover (rank zero, one), while short saddles and small bilobates with concave ends are more frequent in areas of increased plant cover (rank two, three). However, Chi-square testing shows no significant change in the relative frequencies of short cell morphotypes related to plant cover today. In fact, where one phytolith type increases in frequency, so do the other two: These morphotypes vary positively with each other.

### Plant phytoliths

#### Production

**Figure 7:**
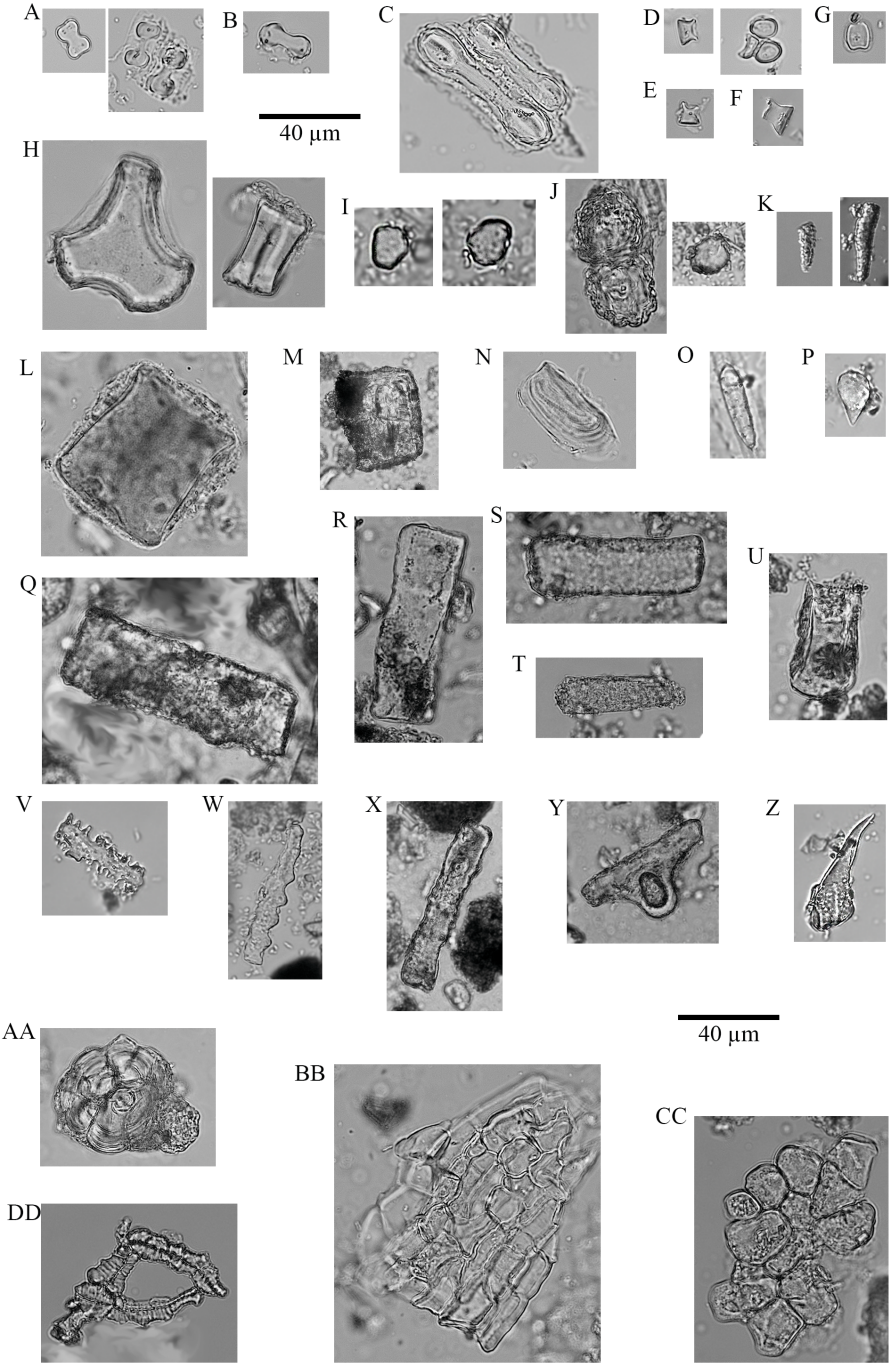
Plant phytoliths. (A) bilobate, short concave (left: *P. mezianum* right: *A. adoensis*); (B) bilobate, short convex (*A. adoensis*); (C) bilobate, long convex (*A. adoensis*); (D) rondel, ovate (*S. panicoides*); (E) rondel, horned (*S. spp.*); (F) tower (*S. consimilis*); (G) saddle, short (*C. dactylon*); (H) bulliform (*S. consimilis*); (I) globulose (*A. secundiflora*); (J) globular granulate (left: *B. eranthemoides* right: *A. secondiflora*); (K) oblong granulate (left: *B. angustifolia*; right: *H. forskaolii*); (L) blocky (*Ocimum spp.*); (M) blocky (*A. tortilis*); (N) blocky radiating (*X. caffra*); (O) fusiform (*O. spp.*); (P) guttiform (*C. cactiformis*); (Q) tabular sinuate (*A. mellifera*); (R) tabular psilate (*A. mellifera*); (S) tabular (*A. tortilis*); (T) tabular scrobiculate (*M. tryphilla*); (U) tabular strangulated (*S. persica*); (V) tabular crenate (*S. consimilis*); (W) ** cylinder sinuate (*A. nilotica*); (X) cylinder scrobiculate (*C. cactiformis*); (Y) sclereid (*A. tortilis*); (Z) hair (*C. dactylon*); (AA) hair base (*X. caffra*) (BB) epidermal cells (*B. eranthemoides*); (CC) globular mesophyll (*X. caffra*); DD) vessel (*A. tortilis*).

All plant samples contained phytoliths, except for the woody tissue of *Commiphora kua*. A total of 4,310 phytoliths were classified (Tables 5, 6) in morphotypes (*n* = 52) and classes (*n* = 13). The median number of phytoliths per species is 130 (range: zero –539) and were found mostly in leaf material (62.01%) with specimens from woody tissue making up the second largest group (23.52%) (Fig. 7, Table S1). The number of samples from leaf and woody tissue was identical at nineteen each. Leaf samples produced on average two and a half times as many phytoliths, with the exception of *Balanites aegyptiaca*, *Melhania parviflora*, and *Ocimum* spp. in which woody tissue generates more phytoliths. Where extraction weights of phytoliths can be compared between leaf and woody tissue from the same species (*n* = 13), leaf samples have a slight tendency to produce larger extraction percentages (8/13).

**Table 3:** Woody versus grassy phytoliths per plant cover rank, and short cell breakdown in topsoils.

**Plant cover rank**	**Woody**	**Grass**	**Short cell class**
			**Lobate**	**Rondel**	**Saddle**	**Grand total**
0	74.8	25.2	34.4	36.3	29.3	100.0
1	72.7	27.3	29.0	39.3	31.7	100.0
2	79.3	20.7	35.8	33.3	30.9	100.0
3	80.8	19.2	37.3	33.3	29.4	100.0

**Table 4:** Percent of short cell morphotypes per plant cover rank in topsoils.

**Morphotype**	**Plant cover rank**				**Grand total**
	**0**	**1**	**2**	**3**	
**Most frequent short cell**	
Rondel, tower	36.7	36.9	30.6	31.9	34.6
Saddle, short	33.5	34.1	35.8	32.6	34.2
Bilobate, short concave	29.9	29.0	33.6	35.4	31.2
Grand total	100.0	100.0	100.0	100.0	100.0
**Saddle**	
Saddle, ovate	10.4	0.0	0.6	0.0	3.8
Saddle, short	70.7	71.8	80.2	79.7	74.3
Saddle, squat	18.9	28.2	19.6	21.7	22.1
Grand Total	100.0	100.0	100.0	100.0	100.0
**Rondel**	
Rondel, horned	1.9	0.9	0.6	0.0	1.1
Rondel, tower	62.5	62.6	63.4	68.7	63.3
Rondel, truncated	2.5	0.9	1.1	1.5	1.6
Rondel	33.1	35.6	34.9	29.9	34.0
Grand Total	100.0	100.0	100.0	100.0	100.0
**Bilobate**	
Bilobate, long concave	5.4	4.7	5.9	6.7	5.5
Bilobate, long convex	1.9	0.6	1.1	0.0	1.2
Bilobate, long flat	3.5	1.2	1.1	1.3	2.0
Bilobtae, long notched	1.5	0.0	0.0	0.0	0.6
Bilobate, short concave	53.8	66.9	64.9	68.0	61.6
Bilobate, short convex	8.1	7.6	9.0	9.3	8.3
Bilobate, short flat	21.5	16.3	11.2	12.0	16.4
Bilobate, short notched	3.8	1.7	6.9	2.7	4.0
Cross	0.0	0.6	0.0	0.0	0.1
Polylobate	0.4	0.6	0.0	0.0	0.3
Grand Total	100.0	100.0	100.0	100.0	100.0

**Table 5:** Plant phytolith counts per group, class, and morphotype.

**Major group**	**Phytolith class**	**Phytolith morphotype**	**Count**
Poaceae short cell	Lobate	Bilobate, long concave	26
		Bilobate, long convex	102
		Bilobate, short concave	491
		Bilobate, short convex	30
		Bilobate, short flat	19
		Polylobate	19
	Rondel	Rondel, horned	82
		Rondel, ovate	464
		Rondel, tower	86
		Rondel	11
	Saddle	Saddle, short	211
		Saddle, squat	15
Woody	Blocky	Blocky	79
		Blocky radiating	9
		Blocky scrobiculate	1
	Cylindrical	Cylinder crenate	8
		Cylinder psilate	35
		Cylinder scrobiculate	6
		Cylinder sinuate	46
	Fusiform	Fusiform	16
		Guttiform	23
	Sclereid/Clavate	Clavate	7
		Sclereid	11
	Spherical	Globular granulate	132
		Globular granulate, large	2
		Globular granulate oblong	11
		Globular psilate	1
		Globular tuberculate	5
		Globulose	239
		Hemisphere psilate	1
		Oblong granulate	596
	Tabular	Tabular ellipsoidal	46
		Tabular laminate	4
		Tabular oblong	9
		Tabular scrobiculate	113
		Tabular sinuate	74
		Tabular strangulated	2
		Tabular subrounded	18
		Tabular sulcate	3
		Tabular thick lacunate	12
Undetermined grass	Grass, undetermined	Bulliform	63
		Trichome	19
Rare/Unknown	Composite	Epidermal cells	131
		Vessel	224
	Epidermal complex	Globular mesophyll	127
		Hair	62
		Hair base	104
		Palisade	6
		Stomata	17
	Tabular, undetermined	Tabular	279
		Tabular crenate	54
		Tabular psilate	159

**Table 6:** Count of phytoliths by species and major group in descending order.

**Species**	**Phytolith major group**				**Grand total**
	**Poaceae short cell**	**Rare/ Unknown**	**Undetermined grass**	**Woody**	
*Boscia angustifolia*	0	2	0	537	539
*Pennisetum mezianum*	264	64	6	0	334
*Sporobolus africanus*	302	16	12	0	330
*Aristida adoensis*	186	78	15	1	280
*Sporobolus consimilis*	192	2	43	0	237
*Sporobolus spp.*	207	20	0	0	227
*Cynodon dactylon*	208	6	5	0	219
*Sporobolus panicoides*	197	19	1	0	217
*Ximenia caffra*	0	183	0	22	205
*Melhania parviflora*	0	61	0	122	183
*Acacia tortilis*	0	94	0	75	169
*Maerua tryphilla*	0	66	0	102	168
*Acacia nilotica*	0	96	0	64	160
*Cissus cactiformis*	0	59	0	81	140
*Barleria eranthemoides*	0	85	0	45	130
*Ocimum spp*	0	33	0	89	122
*Commiphora spp.*	0	121	0	0	121
*Hypoestes forskaolii*	0	2	0	107	109
*Aloe secundiflora*	0	3	0	97	100
*Asparagus africanus*	0	54	0	24	78
*Acacia mellifera*	0	12	0	45	57
*Balanites aegyptiaca*	0	23	0	34	57
*Commiphora africana*	0	4	0	25	29
*Cissus quadrangularis*	0	21	0	4	25
*Commiphora merkeri*	0	10	0	13	23
*Sansevieria robusta*	0	13	0	10	23
*Salvadora persica*	0	10	0	6	16
*Sarcostemma viminale*	0	6	0	6	12
Grand Total	1556	1163	82	1509	4310

We do not see a correlation between high phytolith production and the number of morphotypes per taxon (Table 7). The average yield per morphotype is 83 phytoliths. Three types top the highest frequency with figures six times higher than the average: oblong granulate, small bilobate with concave ends, and rondel ovate. In second place, tabular, globulose, vessels, and short saddles. Average richness is noticed for tabular psilate, globular granulates, epidermal cells, hair complex phytoliths, mesophyll cells, and large bilobates with convex ends. Also included in this group are rondel tower, rondel horned, and blocky. All other morphotypes appear at below average frequency. Thus, highly polymorphic species generate average phytolith amounts, and low polymorphic taxa can be top silica accumulators; however, all but one species among the poor phytolith producers (*Balanites aegyptiaca*) fall within the low polymorphism group, probably an artifact of small sample size.

#### Richness

Five taxonomic groups can be distinguished:

1. Top producers rank at median values almost three times higher than the rest of the group (∼332 phytoliths per mount). The lead type is *Boscia angustifolia* (Capparaceae), along with three members of the Poaceae representing three subfamilies, Panicoideae, Chloridoideae, and Aristidoideae. In this area, grassy species produce many more phytoliths than most other plants, outproducing other functional classes by factors ranging between 1.63 and 27.83.
2. High values are also reported for a group of five species with median phytolith number of 218. Four of these species belong with the Chloridoid grasses. One member is with the Olacaceae (*Ximenia caffra*).
3. Average production was noticed in two members of the genus *Acacia* (*tortilis*, *nilotica*) (Fabaceae). Other notable producers include *Melhania* (Malvaceae), *Maerua* (Capparaceae), *Cissus* (Euphorbiaceae), and *Barleria* (Acanthaceae).
4. Low producers with a median of 104 phytoliths include *Ocimum* (Lamiaceae) and *Commiphora* (Burseraceae), and members of the Acanthaceae, Asphodelaceae, and Asparagaceae.
5. Very poor phytolith producers (median value = 19) comprise some of the most characteristic taxa in the region; namely, *Acacia mellifera*, *Balanites aegyptiaca*, three *Commiphora* species, the most emblematic taxon of Oldupai Gorge (*Sansevieria robusta*), *Salvadora persica*, and the genus *Sarcostemma* (Apocynaceae): The lowest absolute rank in the region is that of *Sarcostemma viminale*.

### Morphotypes

The morphotypes from woody plants are led by the globular class through three phytoliths: Oblong shapes with a granulate texture, globulose, and globular granulate. The tabular class includes three morphotypes in abundance: scrobiculate, sinuate, and ellipsoidal; while the cylindrical class peaks with cylinder sinuates and psilates. Blocky morphotypes are relatively common, but four blocky morphotypes could not be found in the local plants we studied: corniculate, ridged, sinuate, and thick lacunate. The Poaceae produce various rondel types, dominated by ovates, towers, and horned towers. Lobate phytoliths comprise six variants but are mostly manifested through two types: small bilobates with concave ends (e.g., Panicoid type) and large bilobates with convex ends (e.g., Aristidoid type). The saddle class comprises two types largely dominated by short saddles both being very common in *Cynodon dactylon*. Several cylindrical phytoliths were documented in thirteen local dicots but were absent or extremely infrequent among the Poaceae: cylindrical crenate, psilate, scrobiculate, and sinuate.

## Discussion

### The Acacia-Commiphora Analog

In East Africa, the transition to arid-adapted woodlands and grasslands over the last two million years has been documented in pedogenic carbonates (Cerling, Bowman & O’Neil, 1988; Levin et al., 2004; Levin et al., 2011; Quinn et al., 2007), mammal, bird, and fish fossils (Bibi & Kiessling, 2015; Bibi et al., 2018; Prassack et al., 2018), micro- and macrobotanical remains like pollen, phytoliths, and wood (Bonnefille, 1982; Bonnefille, 1995; Barboni et al., 1999), and stable isotopes of hydrogen and carbon from leaf wax lipid biomarkers (Magill, Ashley & Freeman, 2013a; Magill, Ashley & Freeman, 2013b; Uno, Polissar & Jackson, 2016; Lupien et al., 2018).

**Table 7:** Plant species studied for phytolith reference, with polymorphism per taxon.

**Clade (Poaceae)**	**Species**	**Morphotype, n**
	*Aloe secundiflora*	8
	*Acacia mellifera*	9
	*Acacia nilotica*	11
	*Acacia tortilis*	20
Aristidoideae	*Aristida adoensis*	15
	*Asparagus africanus*	7
	*Balanites aegyptiaca*	15
	*Barleria eranthemoides*	15
	*Boscia angustiflora*	3
	*Cissus cactiformis*	14
	*Cissus quadrangularis*	6
	*Commiphora africana*	8
	*Commiphora merkeri*	6
	*Commiphora kua*	0
	*Commiphora spp.*	6
Chloridoideae	*Cynodon dactylon*	5
	*Hypoestes forskaolii*	6
	*Maerua triphylla*	11
	*Melhania parviflora*	15
	*Ocimum spp.*	16
Panicoideae	*Pennisetum mezianum*	7
	*Salvadora persica*	6
	*Sansevieria robusta*	7
	*Sarcostemma viminale*	5
Chloridoideae	*Sporobolus africanus*	10
Chloridoideae	*Sporobolus consimilis*	8
Chloridoideae	*Sporobolus panicoides*	4
Chloridoideae	*Sporobolus spp.*	6
	*Ximenia caffra*	14

The phytoliths from present-day vegetation and soil surfaces are the baseline to interpret palaeolandscapes and ecological niches that might have been occupied by early hominins. To study the influence of plant community structure and cover on human ecology, soil phytolith assemblages under known vegetation density and composition can provide a referential to interpret key palaeoecological aspects of early Pleistocene floristic regions and their main vegetation types.

We provide a phytolith analog for *Acacia*-*Commiphora* mosaics that may represent the trend for more open landscapes recorded in East Africa since the early Pleistocene. In this analog, the cohort of phytolith morphologies that identifies semiarid woodland include tabular scrobiculate, tabular sinuate, blocky, globular facetate, tabular thick lacunate, cylinder scrobiculate, tabular sulcate, tower, short saddle, small bilobate with concave ends, and blocky ridged. We did not find unique identifiers or highly diagnostic shapes for the most typical species that today make up the *Acacia*-*Commiphora* ecosystem, but this is hardly surprising and confirms similar observations in adjacent regional centers of plant endemism, such as the Zambezian woodland, where woody dicot types have very little taxonomic power (Mercader et al., 2009) because of redundancy across orders (Bozarth, 1992). Also in common with phytolith spectra from plants and soils in Zambezian woodlands (Mercader et al., 2009; Mercader et al., 2010; Mercader et al., 2011), there is severe overrepresentation of robust morphotypes such as tabulars, blockies, and globulars in addition to lobate short cells.

### Do soil phytoliths mirror physiognomy and composition of vegetation aboveground?

We address this question at two levels, regional (plot = 100 ha) and local (quadrat = 6.25 ha). When considering the larger regional plot, all soil samples are dominated by phytoliths from woody dicots (Table S2– S4) (Albert, Bamford & Cabanes, 2006):

The blocky morphotypes that could not be found in local plants (corniculate, ridged, sinuate, and thick lacunate) were partly tracked by geo-referencing: Blocky ridged and thick lacunate associate with areas of sparse plant growth (Figs. S5–S6). Large numbers of these forms in soils suggest that plants not represented in our inventory might have produced them or that the species producing these types have disappeared in past decades or not seasonally available when we collected our samples.

Cylindrical phytoliths were common in many of the local but were either absent or extremely infrequent among the grasses. Cylinder corniculates and thick lacunates occur only in the soils, while crenates occur only in plants. Cylinder crenates appear in *Acacia mellifera* and *Ocimum* spp. Similarly, the few corniculates recovered from soils may not be inferentially important. However, the relatively large number of cylinder thick lacunates in soils is noteworthy, as plants from the Miombo woodlands (Mercader et al., 2009) produce them. Fusiforms, guttiforms, clavates, and sclereids are found in plants and soils.

The globular class is common in both soil and plant samples. The globular facetate (mesophyll cells) is very abundant, and geo-referencing shows an association with areas covered by plants and absence from bare places (Fig. S7). Two globulose morphotypes were recovered from soils only: globulose bisected and segmented. The former is a unique phytolith that, in adjacent Zambezian woodlands, is restricted to the Solanaceae (Mercader et al., 2009: Fig. 3AE).

Tabular phytoliths are relatively uncommon in the plants but very common in the soils. Four morphotypes could not be found amongst the plants, but were in soil samples: corniculate, ellipsoidal large, facetate, and pilate. Their georeferencing shows no pattern for three of them, but one type (tabular ellipsoidal large) appears solely in locations with plants growing today.

Poaceae phytoliths are skewed in their soil expression (Table 8, Table S5): the local grasses are demonstrated overproducers (Twiss, Suess & Smith, 1969; Novello et al., 2012) but they are not overrepresented in these soils, rather the opposite. We infer that topsoils embody plant communities, past and present, in a time-averaged palimpsest dominated by woodlands (Table S3). It is likely that the heterogenous landscape and the changing grazing history, erosion, and fire regime by pastoralists over the last 60 years (Sinclair et al., 2008) have created a phytolith record of ecological succession underscored by a reversion towards a dominance of woody vegetation, as independently recorded by studies of land cover change (Sinclair et al., 2007; Niboye, 2010) that denote a low human impact on ecosystem dynamics since the 1960s.

**Table 8:** Topsoil and plant phytolith counts per group and class within the 6.25 ha catchment transect.

**Major group**	**Phytolith class**	**Plants *n* = 18**	**Topsoil *n* = 6**
Poaceae short cell	Lobate	169	60
	Rondel	197	61
	Saddle	225	78
Woody	Blocky	69	290
	Cylindrical	65	80
	Fusiform	30	21
	Sclereid/Clavate	11	15
	Spherical	185	59
	Tabular	166	1087
Undetermined grass	Grass, undetermined	21	65
Rare/Unknown	Composite	195	2
	Epidermal complex	201	0
	Tabular, undetermined	263	94

Within the short cells, three major classes were identified in plants and soils: lobates, rondels, and saddles; albeit saddles are overrepresented in soils. Ten phytolith morphotypes were recovered within the lobate class. Of these, six were found in both plant and soil samples and four were found only in soils (small bilobate with notched ends; large bilobate with flat ends; large bilobate with notched ends; cross). The lead lobate type (Fahmy, 2008; Neumann et al., 2017), small bilobate with concave ends, is produced abundantly in one local and one regional taxon: *Penninsetum mezianum* and *Sporobolus africanus* respectively. Aristidoid, large bilobates with convex ends are documented in *Aristida adoensis* while small bilobates with flat ends appear in *Pennisetum mezianum*. Rondel class phytoliths are common in both plants (*Sporobolus* specimens) and soils. Rondel ovates are unique to the plant sample, while truncated rondels occur only in soils. Rondels and horned rondels appear in *Sporobolus* spp. The absence of rondel ovates in the soils is notable given their preponderance in the genus *Sporobolus*. Saddles were recovered from three plants. Saddle squats are present in *Cynodon dactylon* and the abundance of this morphotype in the soil samples may suggest correlation. Short saddles are found in plants and soils while saddle ovates are from soils but not plants. In brief, short cell phytoliths from soils do not always reflect the grass species growing in them today.

### Catchment

We explore the notion of catchment by zooming into one local quadrat covering 6.25 ha to conduct a side-by-side comparison of phytolith assemblages from topsoils (*n* = 6) and plants (*n* = 18) (Table 8, Tables S4– S5). To do this, we tallied all phytoliths by class. Of the thirteen phytolith classes represented in this quadrat, eight classes are severely underrepresented in the soil, including all three Poaceae short cell classes, two woody classes (globular/fusiform), epidermal cells in anatomical connection, and vessels (Bamford, Albert & Cabanes, 2006). Specifically, a positive balance exists for cylindrical shapes from woody tissue, along with sclereids and clavates. Moreover, dicot tabular morphotypes are overrepresented in the soil by a factor of seven. Several blocky morphotypes from woody tissue are four times more common in soils than in the plants that produced them, and lack of correspondence was similarly noticed for bulliforms and lanceolates. We attribute this imbalance to the phytolith signal from grasses being dampened by an overwhelming accumulation of woody phytoliths.

Rich phytolith producers such as the Poaceae display differential representation in soils, especially at sampling loci where they were growing. For instance, *Cynodon dactylon* produces short and squat saddles. In the quadrat where it grows today the average frequency of short saddles is half (*n* = 7.5) of the local average (*n* = 13), and the same applies to squat saddles (this quadrat = 1.8 versus 3.5 in the rest of the collection). *Sporobolus panicoides* preferentially generates rondel ovates, but none could be found in any of the six soil samples surrounding it. *Aristida adoensis* supports a majority of large bilobates with convex edges, and the average representation in the plant (*n* = 0.2) is similar to that in the soils nearby (*n* = 0.3). Importantly, one of the highest ranked short cells from both collections, soils and plants, the small bilobate with concave ends, attains lower than average representation in the soils under scrutiny (*n* = 4.8/6) perhaps because the main producers of this morphotype (*Pennisetum mezianum*, *Sporobolus africanus*) were not close.

Considering that all phytolith classes found in the living tissues of the eighteen plant taxa growing within this quadrat are recorded in the underlying soil surface it can be suggested that the catchment area for these soil phytolith assemblages is smaller than five hectares (Blinnikov, 1994; Fredlund & Tieszen, 1994; Blinnikov, Busacca & Whitlock, 2002).

### Ecological disturbance, time averaging, heterogeneity, and sampling methods

The soil and plant phytolith assemblages are clearly distinguishable from one another. The presence of rare group phytoliths influences ratios in plants (26.98% of the total assemblage) and is much less an aspect of the soils (4.11%). However, even if we were to remove rare and delicate morphotypes, and consider short cells and woody dicot phytoliths only, the differences between the two datasets are obvious. In the plants, the two major groups are near parity (ratio = 1.031; Poaceae *n* = 1, 556 / woody *n* = 1, 509) while in the soils the two groups are quite disparate in relative frequency (ratio = 0.263; Poaceae *n* = 1, 509 and woody *n* = 7, 914).

Soil samples are not homogenous relative to one another. We indicated statistically significant relationships between the soil phytolith population in samples collected under a given plant cover and the rank of that zone, although not all individual samples show this relationship. The ratio between Poaceae short cells and woody phytoliths varies widely from 1:1 to 0:1. Moreover, soil samples with high number of Poaceae short cells have the highest number of woody phytoliths. Even in the most open parts of the landscape phytoliths from woody tissue outnumber those from grasses (Gao et al., 2018a; Gao et al., 2018b) and some woody types abound in soils but are absent from the local plants sampled (Albert, Bamford & Esteban, 2015).

We are suggesting decadal inheritance of soil phytoliths, as evidenced by:

1) The highest phytolith recovery is from areas without plant cover or Rank Zero.
2) No significant change in the relative frequencies of short cell morphotypes related to plant cover today.
3) Multiplicity of soil phytolith types, woody and grassy, that cannot be traced back to local plants.
4) Several phytolith classes are overrepresented in the soils.

That is, although soil phytoliths might represent ‘decay-in-place’ (Piperno, 2006), as well as transport by wind, water, fire and herbivory (Fredlund, Egan & Howell, 2001), the two phytolith populations reported here, from plants and soils, will always differ from each other because soil assemblages are the sum of phytolith accumulations and losses over time (Fredlund & Tieszen, 1994; Kerns, Moore & Hart, 2001).

## Conclusion

Over the last few decades, different environmental proxies have been utilized in Africa to identify present-day ecosystems that could be used as referentials to frame the emergence of the genus *Homo* (Cerling, Bowman & O’Neil, 1988; Barboni et al., 1999; Barboni et al., 2010; Quinn et al., 2007; Albert & Marean, 2012; Barboni, 2014; Esteban et al., 2017; Novello et al., 2018; Prassack et al., 2018). One of these analogs is the *Acacia-Commiphora* biome, where we studied a phytolith reference collection of characteristic plants that aided in the taxonomic identification of phytoliths from the underlying soils. This article also studied the resolution of phytoliths from present-day surfaces along the *Acacia*-*Commiphora* landscape to reflect woody dicot versus grassy plant cover, detecting the shifting boundary between woodlands and grasslands accurately: sparsely vegetated areas supported a larger pool of grass phytoliths while terrain covered by woody taxa yielded more woody phytoliths. In addition, soil phytolith assemblages recorded decadal ecological succession and changes in vegetation communities.

The comparison of soil phytoliths with local plants suggested that differential production, accumulation, and preservation interplay at the soil level. Well-known silica accumulators such as grasses seem localized and minor because of the cyclical, long-term succession to woodland. Although grasses boomed and busted through the waxing and waning of anthropogenic disturbance by fire and grazing, the long-term accumulation of woody types counterbalanced Poaceae representation. Therefore, phytoliths from local soils are capable of identifying woodlands as the climactic land cover across the landscape and they have value as a long-term proxy to detect ecosystem change and regulation that become apparent over a period of decades. The areal catchment recorded in the soils of the eastern Serengeti Plains encompasses several hectares. Furthermore, a heterogeneous record calls for research design that is able to capture spatial variability, documented here through large-scale soil sampling. The identified phytolith analog will let future work along the East African Rift System frame the environmental context of human evolution whenever the phytolith assemblages from associated sediments and paleosols yield a cohort of co-dominant types featuring the phytolith classes from dicot woody tissue and grasses identified here as typical of *Acacia*-*Commiphora* mosaics.

##  Supplemental Information

10.7717/peerj.8211/supp-1 Figure S1 Extraction weight per sample in relation to plant cover rank and altitude

10.7717/peerj.8211/supp-2 Figure S2 Highest ranking rondel phytolith proportions in relation to plant cover

10.7717/peerj.8211/supp-3 Figure S3 Highest ranking bilobate phytolith proportions in relation to plant cover rank

10.7717/peerj.8211/supp-4 Figure S4 Highest ranking saddle phytolith proportions in relation to plant cover rank

10.7717/peerj.8211/supp-5 Figure S5 Topsoil samples with presence of blocky ridged morphotype

10.7717/peerj.8211/supp-6 Figure S6 Topsoil samples with presence of blocky thick lacunate morphotype

10.7717/peerj.8211/supp-7 Figure S7 Topsoil samples with presence of globular facetate morphotype

10.7717/peerj.8211/supp-8 Table S1 Count of phytoliths by modern plant species and plant part

10.7717/peerj.8211/supp-9 Table S2 Topsoil sample ratio of Poaceae short cell versus woody morphotypes in descending order of value

10.7717/peerj.8211/supp-10 Table S3 Major group representation by sample type

10.7717/peerj.8211/supp-11 Table S4 Blocky, cylindrical, spherical, tabular morphotypes by sample origin

10.7717/peerj.8211/supp-12 Table S5 Poaceae short cell group and class morphotypes by sample origin

## Additional Information and Declarations

### Field Study Permissions

The following information was supplied relating to field study approvals (i.e., approving body and any reference numbers): The Tanzania Commission for Science and Technology authorized this work under permit no. 2018-112-NA-2018-36. The Tanzanian Ministry of Natural Resources and Tourism, through its Antiquities Division, granted us permission to carry out this work (14/2017/2018) and authorities at the Ngorongoro Conservation Area allowed us to enter the protected area (BE.504/620/01/53). The export license for the materials presented in this study were obtained from the Antiquities Division (EA.150/297/01: 5/2018/2019) and the Tanzanian Executive Secretary from the Mining Commission (00001258).

### Data Availability

The following information was supplied regarding data availability: Detailed phytolith extraction protocols and full datasets with individual counts for both soils and plants are available at: Department of Anthropology & Archaeology, Tropical Archaeology Laboratory, ”Protocols: extraction of phytoliths from modern plants and sediment, and preparation of sediment for particle size analysis,” 2018, DOI https://dx.doi.org/10.20383/101.0123. Mercader, Julio; Clarke, Siobhan; Bundala, Mariam; Favreau, Julien; Inwood, Jamie; Itambu, Makarius; Larter, Fergus; Lee, Patrick; Garnet Lewiski-McQuaid, Garnet; Mollel, Neduvoto; Mwambwiga, Aloyce; Patalano, Robert; Soto, Maria; Tucker, Laura; Walde, Dale, ”Soil and Plant Phytoliths from the Acacia-Commiphora Mosaics at Olduvai Gorge (Tanzania),” 2018, DOI https://dx.doi.org/10.20383/101.0122.
