# Supplementary material for: Soil and plant phytoliths from the Acacia-Commiphora mosaics at Oldupai Gorge (Tanzania)
Source: PeerJ. 2019 Dec 11;7:e8211. doi: 10.7717/peerj.8211 (PMC6911344; doi:10.7717/peerj.8211)
Supplement: Figure S4 [file peerj-07-8211-s004.pdf]

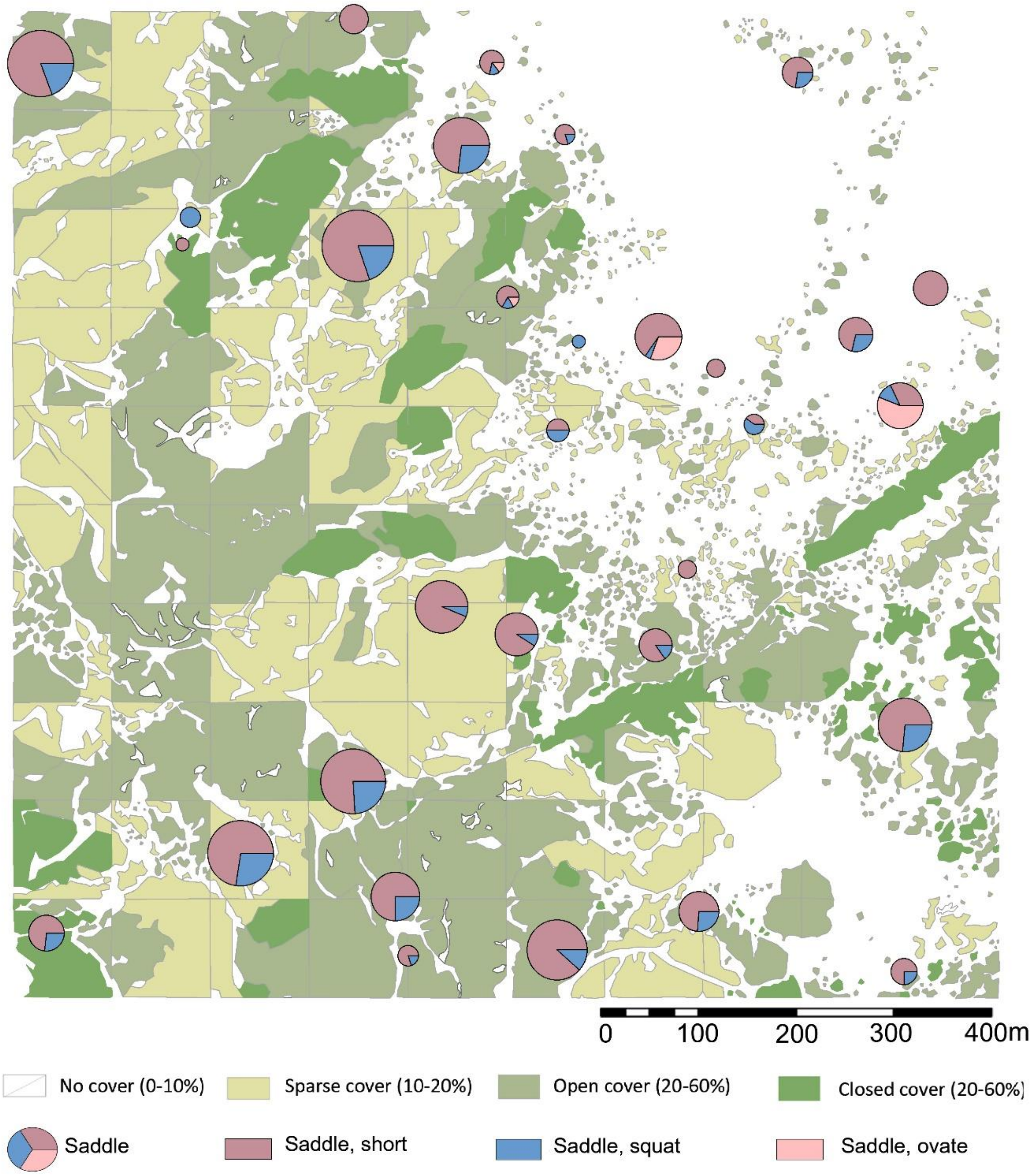

Supplemental Figure 4: Highest ranking saddle phytolith proportions in relation to plant cover rank. Pie size is proportional to phytolith count. For additional information go to doi:10.20383/101.0122
