# Supplementary figures and images for: Soil and plant phytoliths from the Acacia-Commiphora mosaics at Oldupai Gorge (Tanzania)
Source: PeerJ. 2019 Dec 11;7:e8211. doi: 10.7717/peerj.8211 (PMC6911344; doi:10.7717/peerj.8211)

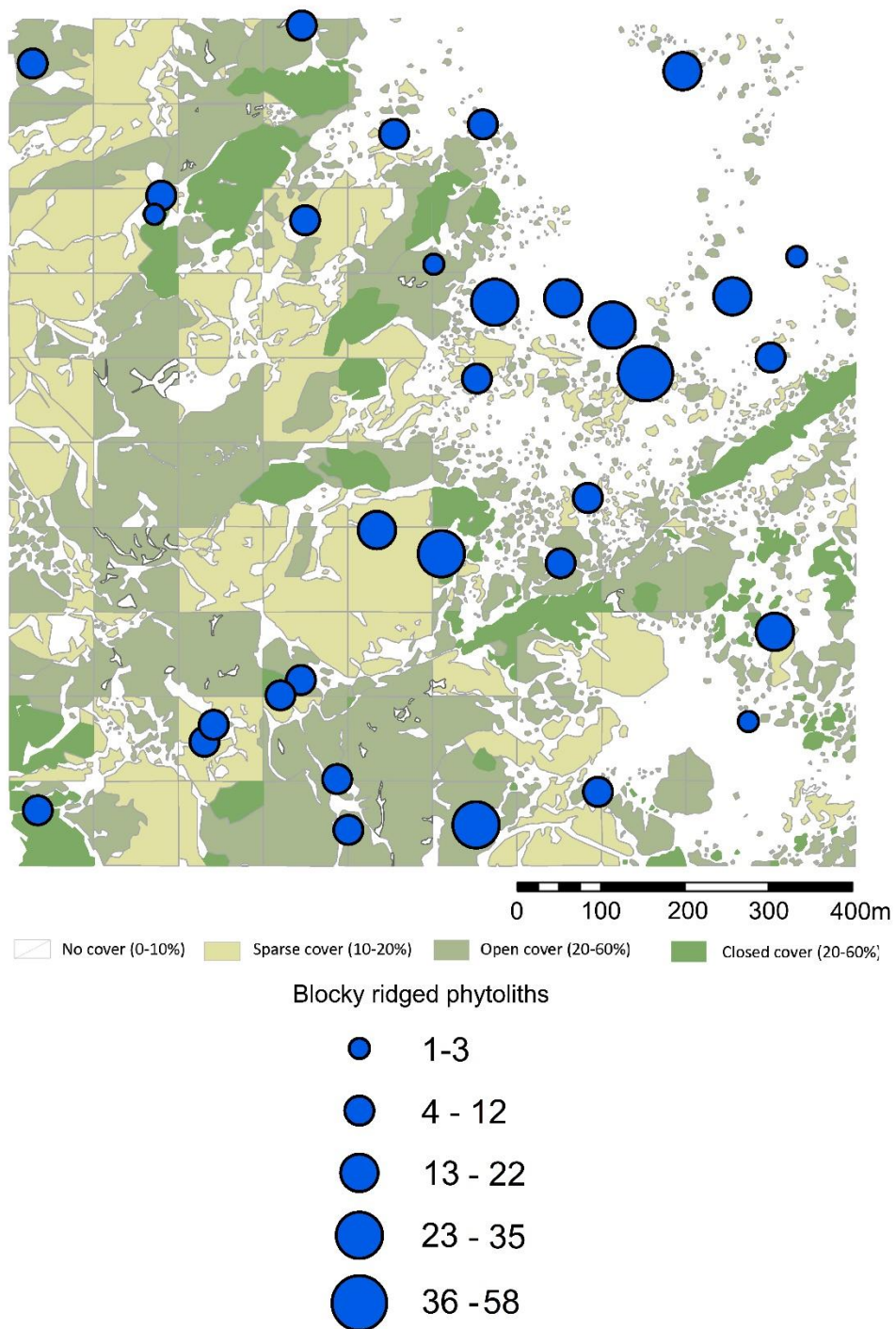

Supplemental Figure 5: Topsoil samples with presence of blocky ridged morphotype.

Supplement: Figure S5 [file peerj-07-8211-s005.pdf]

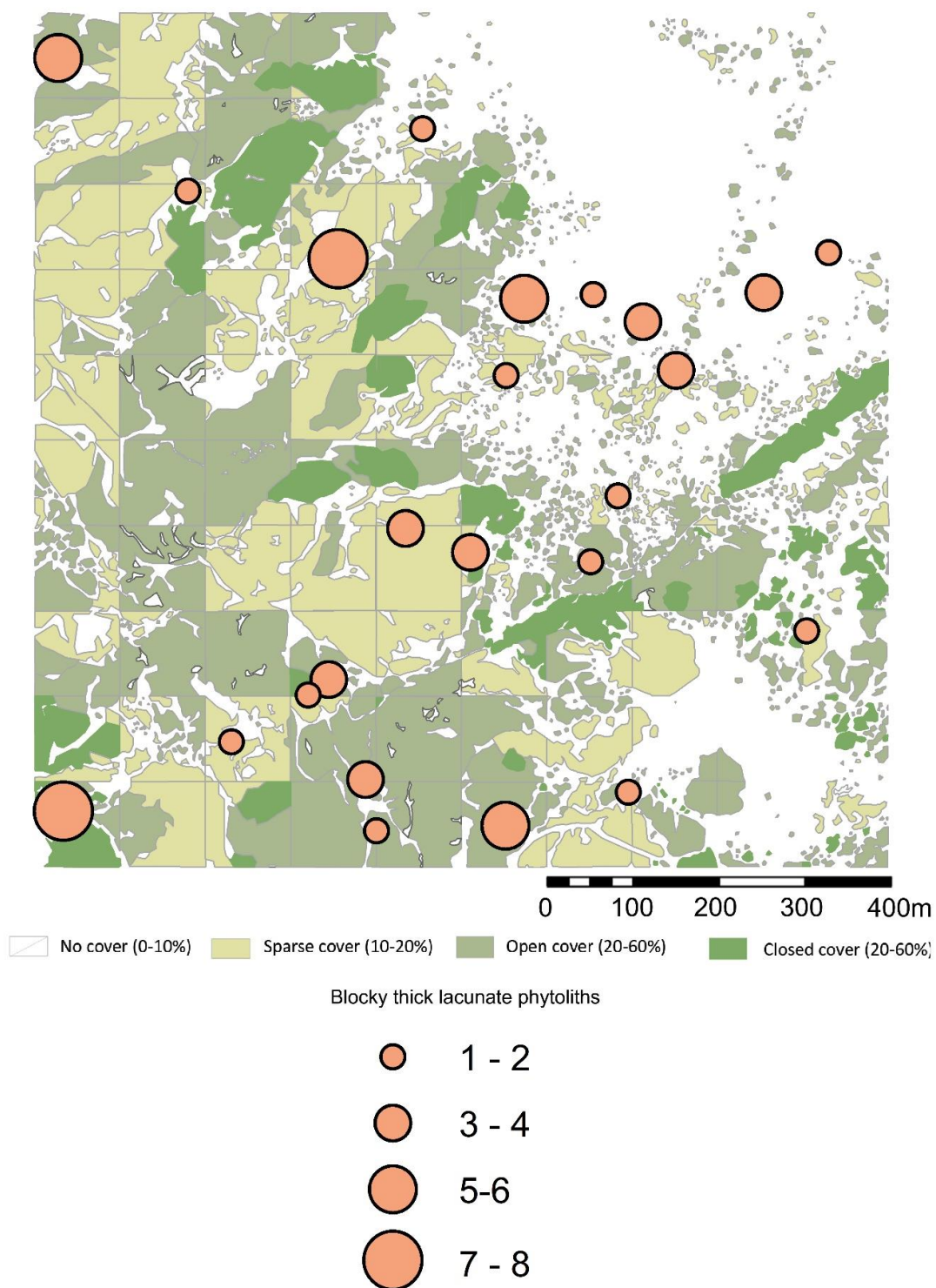

Supplemental Figure 6: Topsoil samples with presence of blocky thick lacunate morphotype.

Supplement: Figure S6 [file peerj-07-8211-s006.pdf]

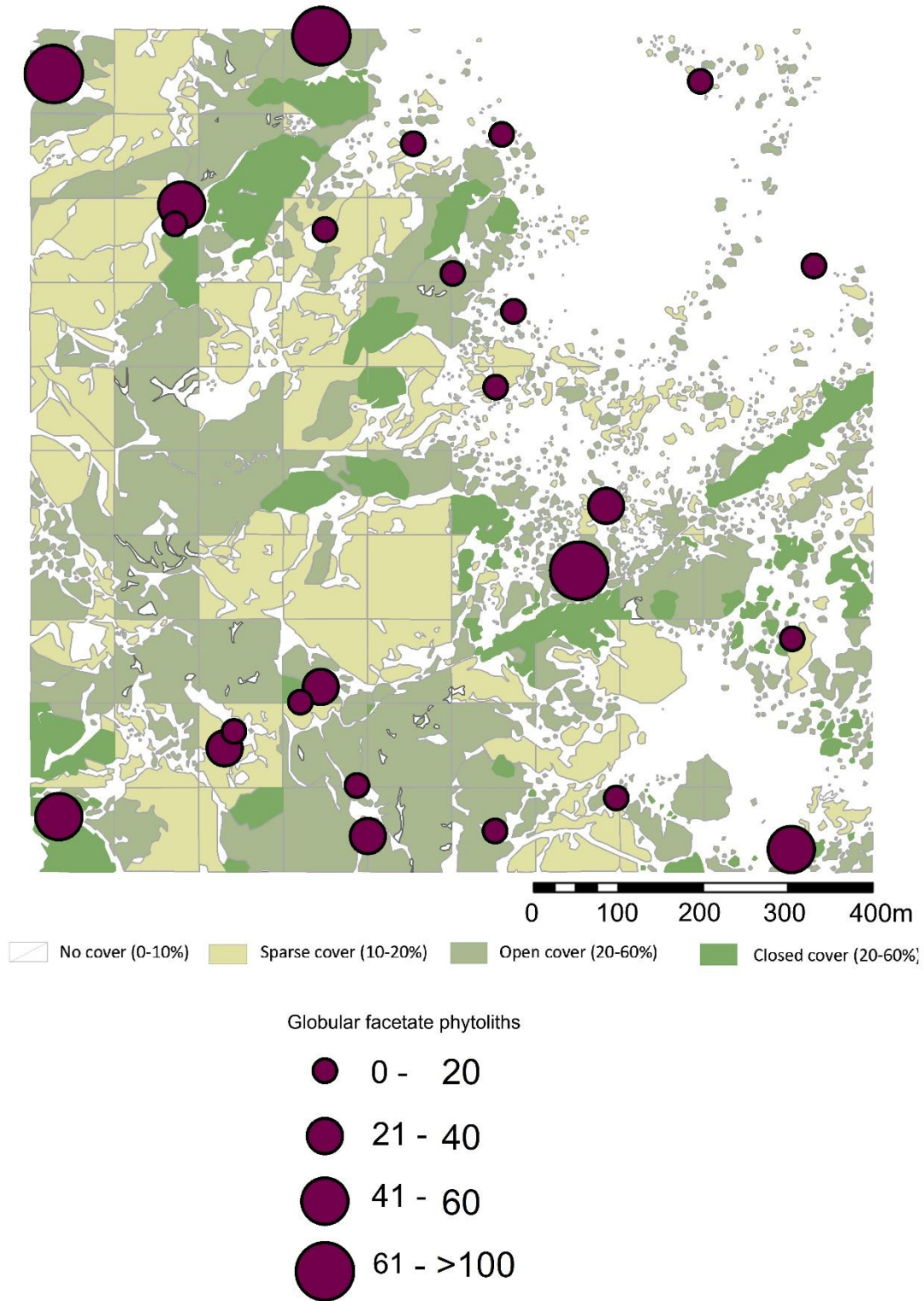

Supplemental Figure 7: Topsoil samples with presence of globular facetate morphotype.

Supplement: Figure S7 [file peerj-07-8211-s007.pdf]
