# Supplementary material for: Soil and plant phytoliths from the Acacia-Commiphora mosaics at Oldupai Gorge (Tanzania)
Source: PeerJ. 2019 Dec 11;7:e8211. doi: 10.7717/peerj.8211 (PMC6911344; doi:10.7717/peerj.8211)
Supplement: Table S1 [file peerj-07-8211-s008.pdf]

Supplemental Table 1: Count of phytoliths by modern plant species and plant part.

**Species**	**Plant Part**					**Grand Total**
	**Culm**	**Leaf**	**Thorn**	**Whole**	**Woody Tissue**	
Acacia mellifera	0	0	0	0	57	57
Acacia nilotica	0	130	0	0	30	160
Acacia tortilis	0	107	0	0	62	169
Aloe secundiflora	0	93	0	0	7	100
Aristida adoensis	0	280	0	0	0	280
Asparagus africanus	0	40	0	0	38	78
Balanites aegyptiaca	0	21	0	0	36	57
Barleria eranthemoides	0	113	0	0	17	130
Boscia angustifolia	0	304	0	0	235	539
Cissus cactiformis	0	0	0	0	140	140
Cissus quadrangularis	0	0	0	0	25	25
Commiphora africana	0	0	0	0	29	29
Commiphora merkeri	0	0	23	0	0	23
Commiphora spp	0	121	0	0	0	121
Cynodon dactylon	0	219	0	0	0	219
Hypoestes forskaolii	0	107	0	0	2	109
Maerua tryphilla	0	109	0	0	59	168
Melhania parviflora	0	44	0	0	139	183
Ocimum spp.	0	44	0	0	78	122
Pennisetum mezianum	50	284	0	0	0	334
Salvadora persica	0	2	0	0	14	16
Sansevieria robusta	0	0	0	0	23	23
Sarcostemma viminale	0	0	0	0	12	12
Sporobolus africanus	0	0	0	330	0	330
Sporobolus consimilis	0	237	0	0	0	237
Sporobolus panicoides	217	0	0	0	0	217
Sporobolus spp.	0	227	0	0	0	227
Ximenia caffra	0	194	0	0	11	205
Grand Total	267	2676	23	330	1014	4310
