# Supplementary material for: Soil and plant phytoliths from the Acacia-Commiphora mosaics at Oldupai Gorge (Tanzania)
Source: PeerJ. 2019 Dec 11;7:e8211. doi: 10.7717/peerj.8211 (PMC6911344; doi:10.7717/peerj.8211)
Supplement: Table S2 [file peerj-07-8211-s009.pdf]

Supplemental Table 2: Topsoil sample ratio of Poaceae short cell versus woody morphotypes in descending order of value.

Sample	Poaceae	Woody	Ratio	Overall Sample Size
112	278	284	0.9789	600
98	197	347	0.5677	573
145	141	262	0.5382	430
65	132	270	0.4889	422
277	102	269	0.3792	388
30	201	596	0.3372	855
258	48	144	0.3333	218
183	70	211	0.3318	304
240	37	125	0.2960	171
176	19	66	0.2879	95
160	84	302	0.2781	415
153	128	473	0.2706	642
131	98	367	0.2670	498
271	28	109	0.2569	146
96	60	267	0.2247	351
292	36	171	0.2105	213
191	56	293	0.1911	356
200	52	301	0.1728	413
54	40	236	0.1695	293
149	18	113	0.1593	151
195	26	164	0.1585	204
172	17	110	0.1545	142
2	40	263	0.1521	350
166	17	120	0.1417	143
260	33	260	0.1269	315
121	14	115	0.1217	140
170	26	232	0.1121	277
66	8	98	0.0816	138
120	24	296	0.0811	339
227	18	246	0.0732	272
230	22	437	0.0503	491
53	4	82	0.0488	96
97	4	89	0.0449	103
245	0	14	0.0000	16
287	0	182	0.0000	185
