# Supplementary material for: Soil and plant phytoliths from the Acacia-Commiphora mosaics at Oldupai Gorge (Tanzania)
Source: PeerJ. 2019 Dec 11;7:e8211. doi: 10.7717/peerj.8211 (PMC6911344; doi:10.7717/peerj.8211)
Supplement: Table S3 [file peerj-07-8211-s010.pdf]

Supplemental Table 3: Major group representation by sample type.

Major Group	Sample Origin		Grand Total
	Modern Plant	Soil	
Poaceae short cell	1556	2078	3634
Rare/Unknown	1163	442	1605
Undetermined Grass	82	311	393
Woody	1509	7914	9423
Grand Total	4310	10745	15055
