# Supplementary material for: Soil and plant phytoliths from the Acacia-Commiphora mosaics at Oldupai Gorge (Tanzania)
Source: PeerJ. 2019 Dec 11;7:e8211. doi: 10.7717/peerj.8211 (PMC6911344; doi:10.7717/peerj.8211)
Supplement: Table S4 [file peerj-07-8211-s011.pdf]

Supplemental Table 4: Blocky, cylindrical, spherical, tabular morphotypes by sample origin.

**Morphotype**	**Sample Origin**		**Grand Total**
	**Modern Plant**	**Soil**	
**Blocky**			
Blocky	79	879	958
Blocky corniculate	0	2	2
Blocky radiating	9	0	9
Blocky ridged	0	405	405
Blocky scrobiculate	1	23	24
Blocky sinuate	0	28	28
Blocky thick lacunate	0	72	72
Grand Total	89	1409	1498
**Cylindrical**			
Cylinder corniculate	0	3	3
Cylinder crenate	8	0	8
Cylinder psilate	35	132	167
Cylinder scrobiculate	6	569	575
Cylinder sinuate	46	115	161
Cylinder thick lacunate	0	61	61
Grand Total	95	880	975
**Spherical**			
Globular bisected	0	1	1
Globular facetate	0	778	778
Globular granulate	132	83	215
Globular granulate large	2	3	5
Globular granulate oblong	11	24	35
Globular psilate	1	10	11
Globular tuberculate	5	0	5
Globulose	239	2	241
Globulose segmented	0	1	1
Hemisphere psilate	1	3	4
Oblong granulate	596	10	606
Grand Total	987	915	1902
**Tabular**			
Tabular corniculate	0	11	11
Tabular ellipsoidal	46	35	81
Tabular ellipsoidal large	0	21	21
Tabular facetate	0	8	8
Tabular laminate	4	186	190
Tabular oblong	9	16	25
Tabular pilate	0	3	3
Tabular scrobiculate	113	2036	2149

Tabular sinuate	74	918	992
Tabular strangulated	2	11	13
Tabular subrounded	18	0	18
Tabular sulcate	3	509	512
Tabular thick lacunate	12	731	743
Grand Total	281	4485	4766
