# Supplementary material for: Soil and plant phytoliths from the Acacia-Commiphora mosaics at Oldupai Gorge (Tanzania)
Source: PeerJ. 2019 Dec 11;7:e8211. doi: 10.7717/peerj.8211 (PMC6911344; doi:10.7717/peerj.8211)
Supplement: Table S5 [file peerj-07-8211-s012.pdf]

Supplemental Table 5: Poaceae short cell group and class morphotypes by sample origin.

**Morphotype**	**Sample Type**		**Grand Total**
	**Modern Plant**	**Soil**	
**Lobate**			
Bilobate, long concave	26	38	64
Bilobate, long convex	102	8	110
Bilobate, long flat	0	14	14
Bilobate, long notched	0	4	4
Bilobate, short concave	491	428	919
Bilobate, short convex	30	58	88
Bilobate, short flat	19	114	133
Bilobate, short notched	0	28	28
Cross	0	1	1
Polylobate	19	2	21
Grand Total	687	695	1382
**Rondel**			
Rondel	11	255	266
Rondel, horned	82	8	90
Rondel, ovate	464	0	464
Rondel, tower	86	475	561
Rondel, truncated	0	12	12
Grand Total	643	750	1393
**Saddle**			
Saddle, ovate	0	24	24
Saddle, short	211	469	680
Saddle, squat	15	140	139
Grand Total	226	633	859
**Poaceae short cell class**			
Lobate	687	695	1382
Rondel	643	750	1393
Saddle	226	633	859
Grand Total	1556	2078	3634
